# Effects of Structural Relaxation of Glass-Forming Melts on the Overall Crystallization Kinetics in Cooling and Heating

**DOI:** 10.3390/e25111485

**Published:** 2023-10-26

**Authors:** Jürn W. P. Schmelzer, Timur V. Tropin, Christoph Schick

**Affiliations:** 1 Institut für Physik, Universität Rostock, Albert-Einstein-Strasse 23-25, 18059 Rostock, Germany; christoph.schick@uni-rostock.de; 2 Competence Centre CALOR, Faculty of Interdisciplinary Research, University of Rostock, Albert-Einstein-Str. 25, 18051 Rostock, Germany; 3 BCMaterials, Basque Center for Materials, Applications and Nanostructures, UPV/EHU Science Park, 48940 Leioa, Spain; timur.tropin@bcmaterials.net; 4 Frank Laboratory of Neutron Physics, Joint Institute for Nuclear Research, ul. Joliot-Curie 6, 141980 Dubna, Russia

**Keywords:** nucleation, thermodynamics of nucleation, general theory of phase transitions, theory and models of crystal growth, 64.60.Bd, 64.60.Q, 81.10.Aj, 82.60.Nh

## Abstract

In the theoretical treatment of crystallization, it is commonly assumed that the relaxation processes of a liquid proceed quickly as compared to crystal nucleation and growth processes. Actually, it is supposed that a liquid is always located in the metastable state corresponding to the current values of pressure and temperature. However, near and below the glass transition temperature, $T_{g}$, this condition is commonly not fulfilled. In such cases, in the treatment of crystallization, deviations in the state of the liquid from the respective metastable equilibrium state have to be accounted for when determining the kinetic coefficients governing the crystallization kinetics, the thermodynamic driving force of crystallization, and the surface tension of the aggregates of the newly evolving crystal phase including the surface tension of critical clusters considerably affecting the crystal nucleation rate. These factors may greatly influence the course of the overall crystallization process. A theoretical analysis of the resulting effects is given in the present paper by numerical solutions of the J(ohnson)–M(ehl)–A(vrami)–K(olmogorov) equation employed as the tool to model the overall crystallization kinetics and by analytical estimates of the crystallization peak temperatures in terms of the dependence on cooling and heating rates. The results are shown to be in good agreement with the experimental data. Possible extensions of the theory are anticipated and will be explored in future analysis.

## 1. Introduction

In materials science, the understanding of the kinetics of phase transformation processes, under varying external and/or internal conditions, is of significant importance. One example in this respect consists of the widely studied process of phase formation in glass-forming liquids under cooling and heating by differential scanning calorimetry and differential thermal analysis methods or fast scanning calorimetry [1,2,3]. In the respective experimental investigations, primarily the latent heat of crystallization, melting is measured. Different attempts have been developed to interpret the experimental data theoretically and to derive conclusions concerning the dependence of the nucleation and growth rates on temperature. These studies are of fundamental importance for the understanding of the glass-forming ability of a given substance or for the specification of the conditions that have to be fulfilled to create materials with a well-defined fraction, shape, and distribution of the crystalline phases possibly evolving in them.

One of the most appropriate methods in this analysis consists of the application of the J(ohnson)–M(ehl)–A(vrami)–K(olmogorov) equation (see e.g., [4,5,6]). It is derived by modeling the basic features of crystallization, i.e., the kinetics of nucleation and of the further growth of the supercritical clusters. These processes depend (at constant pressure) on temperature, and their interplay determines the degree of overall crystallization. They are affected significantly by the rates of cooling and heating. For this reason, a variety of investigations have been devoted to it [7,8,9,10,11,12,13,14,15,16]. Since the JMAK equation is a consequence of modeling the interplay of the basic processes determining crystallization, we consider it as being advantageous over other approaches, for example, the Kissinger equation widely employed in the analysis of crystallization [17,18,19,20]. For this reason, we perform our present analysis utilizing the JMAK equation. The main purpose of our study is described as follows.

In the application of classical nucleation theory to the theoretical description of the crystallization of liquids and glasses, it is as a rule assumed that nucleation and the subsequent growth of the crystal phase proceed only after the supercooled liquid or the glass have completed structural relaxation processes towards the corresponding metastable equilibrium state. Only by employing such an assumption can the thermodynamic driving force of crystallization and the surface tension be determined in the way it is commonly performed. However, as shown in detail in the first special issue on “Crystallization Thermodynamics” in Entropy in [21], near and below the glass transition temperature, a different situation is observed as a rule. In this temperature range, these processes proceed concomitantly with structural relaxation. As a consequence, nucleation and growth rates depend not only on temperature but also on the current state of the relaxing melt. A similar behavior is expected to occur if the glass transition is governed by variations in pressure or other external control parameters.

To treat the nucleation kinetics theoretically for such cases, adequate expressions for the thermodynamic driving force and the surface tension are required that account for the contributions caused by the deviation of the supercooled liquid from metastable equilibrium. Utilizing the approach developed by de Donder (see, e.g., [5,6]), these deviations may be expressed via differences of a set of appropriately chosen structural order parameters from their equilibrium values as anticipated for the first time in [22]. Relaxation processes result in changes in the structural order parameters with time. As a consequence, the thermodynamic driving force and the surface tension, and other basic characteristics of crystal nucleation, such as the work of critical cluster formation and the steady-state nucleation rate, as well as the rates of growth, also become time-dependent. The correct description of the relaxation of the structural order parameters is consequently a prerequisite of a correct treatment of nucleation and growth in such cases. As shown in the above-cited paper [21] and in [23] based on the analysis of experimentally observed nucleation rate data, temporarily, the liquid may even be trapped in this relaxation process in local minima of the potential energy landscape resulting in a step-wise change in the work of critical cluster formation and the steady-state nucleation rate (see also [24,25,26,27]). Consequently, the shape of the potential energy landscape and its change with temperature may significantly affect crystal nucleation.

The above-described scenario of nucleation and growth—the interplay of structural relaxation and crystallization—is realized if diffusion (or other appropriate kinetic mechanisms controlling nucleation and growth) and viscosity (responsible widely for the α–relaxation process in the liquid) decouple. Such decoupling occurs as a rule at temperatures $T_{d}$ near to the conventionally defined glass transition temperature, $T_{g}$. Below $T_{d}$, elastic stresses evolving in nucleation and growth may also significantly affect the crystallization kinetics. Consequently, a comprehensive theoretical description of crystal nucleation and growth near and below the glass transition range has to account appropriately for the effects of deviations of the liquid from the metastable states and of their relaxation on the crystal nucleation and growth of crystals in glass-forming liquids. In addition, in this temperature range ($T\leq T_{d}$), the effects caused by simultaneous stress evolution and stress relaxation on crystal nucleation and growth have to be taken into consideration [23,28,29,30,31,32].

These theoretical concepts have been successfully applied to the interpretation of experimental data on nucleation as shown, e.g., in [23,33,34,35] and in another contribution to the present special issue [36]. They also allow a new approach to the understanding of hysteresis effects in crystallization in cooling and heating as partly discussed already in [37]. In the latter mentioned paper, we drew attention to the dependence of the steady-state nucleation rate on the interplay of relaxation and crystal nucleation in cooling and heating. Here, this analysis is extended to the description of the effect of deviations of the glass-forming liquid from metastable equilibrium and its relaxation on the kinetics of overall crystallization in cooling and heating, going considerably beyond the analysis of the effects of the interplay of relaxation and crystallization on the overall crystallization under isothermal conditions performed in [34,35].

This paper is structured as follows. In Section 2, the basic theoretical relations employed for the analysis are summarized. The JMAK equation is briefly developed and the expressions for the thermodynamic and kinetic parameters utilized in the numerical computations are summarized. Next, possible generalizations of the theoretical treatment are listed that need to be accounted for in future studies. Some of these are discussed in detail in the appendices. The results of the numerical solutions of the JMAK equation are given in Section 3. A theoretical analysis of the obtained results is outlined in Section 4. It includes the derivation of analytical estimates of the crystallization peak temperatures in heating and cooling based on the JMAK formalism. A summary of the results and their discussion given in Section 5 completes the paper.

## 2. Basic Equations

### 2.1. Johnson–Mehl–Avrami–Kolmogorov (JMAK) Equation

The degree of overall crystallization, $\alpha_{n}\left(t\right)$, at time, *t*, is defined as the ratio

(1) $$\alpha_{n}\left(t\right)=\frac{V_{n}\left(t\right)}{V},$$

where $V_{n}\left(t\right)$ is the volume crystallized at time *t*, and *V* is the initial volume of the melt at time t=0. The Johnson–Mehl–Avrami–Kolmogorov (JMAK) equation describes the evolution of $\alpha_{n}\left(t\right)$ with time. The origin of this relation can be described as follows.

In the interval of time $\left(t^{\prime },t^{\prime }+dt^{\prime }\right)$ the number

(2) $$dN\left(t^{\prime }\right)=\left(V-V_{n}\left(t^{\prime }\right)\right)J\left(t^{\prime }\right)dt^{\prime }$$

of clusters is formed, which may grow up to macroscopic dimensions. Here, $J(t^{\prime })$ is the nucleation rate per unit volume at time $t^{\prime }$. The clusters are assumed to grow in *n* independent spatial directions with a linear growth rate $u_{i}\left(t^{\prime \prime }\right)$, which may also depend, in general, on time. The contribution to the volume of the new phase, $v(N=1,t\geq t^{\prime })$, originating from one cluster (N=1) formed at the moment $t=t^{\prime }$ at later times $t>t^{\prime }$ is then given by

(3) $$v\left(N=1,t\geq t^{\prime }\right)=\omega_{n}\int_{t^{\prime }}^{t}u_{1}\left(t^{\prime \prime }\right)dt^{\prime \prime }\times \int_{t^{\prime }}^{t}u_{2}\left(t^{\prime \prime }\right)dt^{\prime \prime }\times \ldots \times \int_{t^{\prime }}^{t}u_{n}\left(t^{\prime \prime }\right)dt^{\prime \prime }$$

being equal to

(4) $$v\left(N=1,t\geq t^{\prime }\right)=\omega_{n}{\left(\int_{t^{\prime }}^{t}u\left(t^{\prime \prime }\right)dt^{\prime \prime }\right)}^{n}$$

for growth with the same rate, *u*, in *n* independent perpendicular directions.

In any case, one has to substitute here the volume of the new phase, which evolves due to the growth from one supercritical nucleus formed at $t=t^{\prime }$. This condition specifies the value of the shape factor, $\omega_{n}$, correlating growth rates, and volume of the newly evolving phase. For three-dimensional growth in a radial direction, we obtain as a special case

(5) $$v\left(N=1,t\geq t^{\prime }\right)=\omega_{n}{\left(\int_{t^{\prime }}^{t}u_{R}\left(t^{\prime \prime }\right)dt^{\prime \prime }\right)}^{3},\;u_{R}\left(t\right)=\frac{dR(t)}{dt},\;\omega_{n}=\frac{4\pi }{3},$$

i.e., here, $v(N=1,t\geq t^{\prime })$ is equal to $v\left(N=1,t\geq t^{\prime }\right)=\left(4\pi /3\right)R^{3}\left(t\right)$. Here, and in most other applications, it is commonly assumed that the initial size of the clusters is small as compared with the characteristic sizes at time *t*, i.e., it is supposed that $R^{3}\left(t\right)\gg R^{3}\left(t^{\prime }\right)≅R_{c}^{3}\left(t^{\prime }\right)$, where $R_{c}\left(t^{\prime }\right)$ is the current value of the critical cluster radius.

The total amount of the new phase formed by the $dN(t^{\prime })$ clusters nucleating in the interval $\left(t^{\prime },t^{\prime }+dt^{\prime }\right)$ is given, consequently, by

(6) $$dV_{n}\left(t^{\prime }\right)=dN\left(t^{\prime }\right)v\left(N=1,t\geq t^{\prime }\right)=\left(V-V_{n}\left(t^{\prime }\right)\right)J\left(t^{\prime }\right)dt^{\prime }v\left(N=1,t\geq t^{\prime }\right)$$

or by

(7) $$dV_{n}\left(t^{\prime }\right)=\omega_{n}J\left(t^{\prime }\right)\left(V-V_{n}\left(t^{\prime }\right)\right)dt^{\prime }{\left(\int_{t^{\prime }}^{t}u\left(t^{\prime \prime }\right)dt^{\prime \prime }\right)}^{n}.$$

This equation can be rewritten in the form

(8) $$d\alpha_{n}\left(t^{\prime }\right)=\left(1-\alpha_{n}\left(t^{\prime }\right)\right)dY_{n}\left(t^{\prime }\right),$$

(9) $$dY_{n}\left(t^{\prime }\right)=\omega_{n}J\left(t^{\prime }\right)dt^{\prime }{\left(\int_{t^{\prime }}^{t}v\left(t^{\prime \prime }\right)dt^{\prime \prime }\right)}^{n}.$$

Since clusters are formed in the time interval (0,t), we have to take—in order to determine the fraction of the crystalline phase at time *t*—the sum (integral in the range (0,t)) resulting in Equations (10) and (11),

(10) $$\alpha_{n}\left(t\right)=1-\exp\left[-Y_{n}\left(t\right)\right],$$

(11) $$Y_{n}\left(t\right)=\omega_{n}\int_{0}^{t}J\left(t^{\prime }\right)dt^{\prime }{\left(\int_{t^{\prime }}^{t}u\left(t^{\prime \prime }\right)dt^{\prime \prime }\right)}^{n}.$$

In this integration procedure, it is assumed that both $\alpha_{n}$ and $Y_{n}$ are equal to zero at t=0.

These general relations can be further specified considering particular nucleation and growth modes. We briefly analyze here two special cases employed later in the theoretical analysis of overall crystallization in cooling and heating. One of the simplest cases we are encountered with is if nucleation and growth rates are considered as constant, i.e.,

(12) J(t)=J=constant,u(t)=u=constant.

Under such a condition, the extended volume becomes equal to

(13) $$Y_{n}\left(t\right)=\omega_{n}Ju^{n}\int_{0}^{t}{\left(t-t^{\prime }\right)}^{n}dt^{\prime },$$

leading after integration to the well-known classical result

(14) $$\alpha_{n}\left(t\right)=1-\exp\left(-\frac{\omega_{n}}{\left(n+1\right)}Ju^{n}t^{n+1}\right).$$

A derivation of Equation (14) with respect to time gives the rate of overall crystallization in the form

(15) $$\frac{d\alpha_{n}\left(t\right)}{dt}=k_{n}\left(n+1\right)t^{n}\exp\left(-k_{n}t^{n+1}\right)\;\mathrm{with}\;k_{n}=\frac{\omega_{n}}{\left(n+1\right)}Ju^{n}.$$

The parameter $k_{n}$ is the so-called Avrami kinetic coefficient. Employing this notation, Equation (14) obtains the form

(16) $$\alpha_{n}\left(t\right)=1-\exp\left(-k_{n}t^{n+1}\right).$$

This relation may be utilized to replace time in Equation (15) via the relations

(17) $$\exp\left(-k_{n}t^{n+1}\right)=1-\alpha ,\;t=\frac{1}{k_{n}^{1/n+1}}{\left[-\ln(1-\alpha_{n})\right]}^{1/n+1}.$$

Equation (15) can be rewritten as

(18) $$\frac{d\alpha_{n}\left(t\right)}{dt}=\left(n+1\right)k_{n}^{1/(n+1)}f\left(\alpha_{n}\right),$$

(19) $$f\left(\alpha_{n}\right)=\left(1-\alpha_{n}\right){\left[-\ln\left(1-\alpha_{n}\right)\right]}^{n/(n+1)}.$$

The rate of change in the degree of overall crystallization, $d\alpha_{n}\left(t\right)/dt$, is equal to zero at both limits $\alpha_{n}=0$ and $\alpha_{n}=1$; it has a maximum (for n>1) at a definite value of $\alpha_{n}$ given by d(dα/dt)/dt=0, and it corresponds to the point of inflexion in the $\alpha_{n}\left(t\right)$-curves.

As a second special case, we consider again nucleation–growth under isothermal conditions, so that the growth rate can be taken as constant. However, nucleation is assumed now to proceed exclusively at a number $N_{het}$ of heterogeneous nucleation cores. The nucleation rate at time $t^{\prime }$ can then be expressed as

(20) $$dN\left(t^{\prime }\right)=J_{het}\left(t^{\prime }\right)dt^{\prime }=N_{het}\left(t\right)J_{het}^{0}dt^{\prime }=\left(N_{het}\left(0\right)-N\left(t^{\prime }\right)\right)J_{het}^{0}dt^{\prime }.$$

Here, $J_{het}^{0}$ is a combination of kinetic and thermodynamic parameters describing the considered heterogeneous nucleation process. It is widely independent of time. The solution of this equation is

(21) $$\frac{\left(N_{het}\left(0\right)-N\left(t^{\prime }\right)\right)}{N_{het}\left(0\right)}=\exp\left(-J_{het}^{0}t^{\prime }\right),$$

resulting in

(22) $$J_{het}\left(t^{\prime }\right)=J_{het}\left(0\right)\exp\left(-J_{het}^{0}t^{\prime }\right).$$

In the limiting case of large values of $J_{het}^{0}$, we may express the nucleation rate in the form

(23) $$J_{het}\left(t^{\prime }\right)=N_{het}\left(0\right)\delta \left(t^{\prime }\right),$$

where δ(t) is the Dirac delta function. A substitution into Equation (11) yields

(24) $$Y_{n}\left(t\right)=\omega_{n}N_{het}\left(0\right){\left(\int_{0}^{t}u\left(t^{\prime \prime }\right)dt^{\prime \prime }\right)}^{n}.$$

and for constant values of the growth rate

(25) $$\alpha_{n}\left(t\right)=1-\exp\left(-\omega_{n}u^{n}N_{het}\left(0\right)t^{n}\right).$$

This equation can be rewritten in the form of Equation (16) again, but with the replacements

(26) $$k_{n}=\omega_{n}u^{n}N_{het}\left(0\right),\;n+1\to n.$$

Equations (17)–(19) retain their validity once these replacements are made there.

Note that, accounting for the interplay of relaxation and crystal nucleation and growth, even at isothermal conditions, the general relations, Equations (10), (11), and (24), have to be employed, as a rule, for the description of overall crystallization since the nucleation and growth rates may vary with time due to relaxation. The simple relations, Equations (14) and (25) as described above, are not applicable any more (see also [34,35,36]).

### 2.2. Description of the Rates of Nucleation and Growth and the Kinetics of Relaxation

In order to utilize the JMAK equation, appropriate expressions for the rates of nucleation and growth have to be known. In their specification, we employ the methods discussed in a comprehensive form in [21,37]. Here we reproduce the main results directly required for the computations referring to cited papers for details.

The kinetics of nucleation, we describe by the steady-state nucleation rate, *J*, in the conventional form

(27) $$J=J_{0}\exp\left(-\frac{W_{c}}{k_{B}T}\right),\;W_{c}=\frac{1}{3}\sigma A_{c},\;A_{c}=4\pi R_{c}^{2},\;R_{c}=\frac{2\sigma }{\Delta g}.$$

Here, $W_{c}$ is the work of critical cluster formation, σ the surface tension of crystallites of critical size, $A_{c}$ and $R_{c}$ are the surface area and the radius of the critical cluster modeled to be of spherical shape, respectively, Δg is the change in the bulk contributions to the Gibbs free energy per unit volume of the crystal phase when the metastable liquid is transformed into the crystal, $k_{B}$ is the Boltzmann constant, and *T* the absolute temperature.

Deviations in the state of the liquid from metastable equilibrium are described by a structural order parameter, ξ. Its equilibrium values in the corresponding metastable state are denoted by $\xi_{e}$. Deviations from equilibrium are also expressed by the reduced variable

(28) $$\tilde{\xi }=\frac{\left(\xi -\xi_{e}\right)}{\xi_{e}}.$$

Utilizing a simple lattice-hole model for the description of the configurational contributions to the thermodynamic quantities, the equilibrium value of the structural order parameter is given by

(29) $$\frac{{\left(1-\xi_{e}\right)}^{2}}{\ln\xi_{e}}=-\frac{1}{\chi }\left(\frac{T}{T_{m}}\right).$$

**Figure 1:**
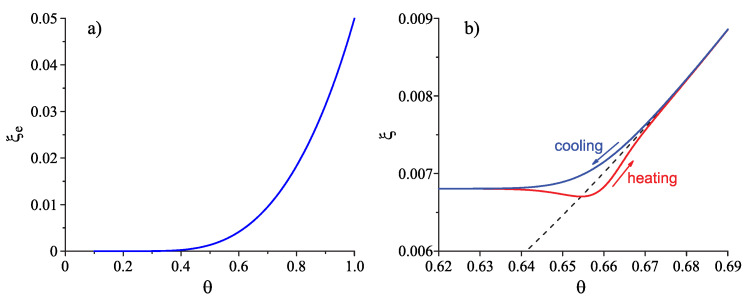
Structural order parameter, ξ, and its equilibrium value, $\xi_{e}$, in terms of dependence on reduced temperature, $\theta =T/T_{m}$. (**a**) Dependence of the equilibrium value of the structural order parameter for the whole range of temperatures between melting or liquidus temperature, $T_{m}$, and absolute zero as obtained in the framework of the lattice model employed here. (**b**) Typical behavior of the structural order parameter, ξ, in dependence on temperature in the vicinity of the glass transition range if the liquid is cooled down and heated with the same constant rate of change in temperature. The dependencies ξ(T) are shown by full curves if the system is cooled down (blue curve) and heated (red curve) with a constant rate (here taken as equal to (dT/dt)=1.3 K/s or $\left(d\theta /dt\right)=10^{-3}s^{-1}$); the dashed curve shows the equilibrium value of this parameter in the given range of temperature. The figure is taken from [21] (Creative Commons Attribution License).

Here, $T_{m}$ is the melting temperature, and the parameter χ we set to equal χ=3.32 again. The dependence of $\xi_{e}$ on the reduced temperature, $\theta =T/T_{m}$, is shown in Figure 1a; the method of determination of the temperature dependence of ξ (typically of the form as shown in Figure 1b) is discussed below.

Accounting for deviations from metastable equilibrium, the work of critical cluster formation can be written in this case as

(30) $$W_{c}\left(T,p;\xi \right)=\Delta G\left(n_{c}\right)=\frac{16\pi }{3}\frac{\sigma^{3}\left(T,p;\xi \right)}{{\left(c\Delta \mu \left(T,p;\xi \right)\right)}^{2}},\;c=\frac{1}{d_{0}^{3}},$$

where $d_{0}$ is a characteristic size parameter determined by the particle number density, *c*. The dependence of the surface tension and the thermodynamic driving force of the crystallization process is given by

(31) $$\Delta \mu \left(T,p_{m};\xi \right)≅\Delta h_{m}\left(1-\frac{T}{T_{m}}\right)\left[1-\frac{\Delta c_{p}}{2\Delta s_{m}}\left(1-\frac{T}{T_{m}}\right)\right]+\frac{k_{B}T\xi_{e}}{2}{\tilde{\xi }}^{2},$$

(32) $$\frac{\sigma (T,p_{m},\xi )}{\sigma (T_{m},p_{m})}=\frac{T}{T_{m}}\left[1-\frac{\Delta c_{p}}{\Delta s_{m}}\left(1-\frac{T}{T_{m}}\right)-\left(\frac{k_{B}\xi_{e}\ln\xi_{e}}{\Delta s_{m}}\right)\tilde{\xi }\right].$$

Here, $\Delta s_{m}$ and $\Delta h_{m}=T_{m}\Delta s_{m}$ are the melting entropy and melting enthalpy, respectively, per particle of the crystal at the temperature, $T_{m}$, and the pressure, $p_{m}$, corresponding to the melting point of the substance; $\Delta c_{p}$ is the difference in the specific heat per particle in the liquid and the crystalline phases, respectively, and also at ($T_{m},p_{m}$). The pressure we take as being constant and equal to the atmospheric pressure.

With Equations (28), (31), and (32), the work of critical cluster formation can be written generally as

(33) $$W_{c}\left(T,p_{m};\xi \right)=W_{c}\left(T,p_{m};\xi_{e}\right)\Theta ,$$

(34) $$\Theta =\frac{{\left\{1-\frac{\left(\frac{k_{B}\xi_{e}\ln\xi_{e}}{\Delta s_{m}}\right)\tilde{\xi }}{\left[1-\frac{\Delta c_{p}}{\Delta s_{m}}\left(1-\frac{T}{T_{m}}\right)\right]}\right\}}^{3}}{{\left\{1+\frac{\left(\frac{k_{B}T\xi_{e}}{2}\right){\tilde{\xi }}^{2}}{\Delta h_{m}\left(1-\frac{T}{T_{m}}\right)\left[1-\frac{\Delta c_{p}}{2\Delta s_{m}}\left(1-\frac{T}{T_{m}}\right)\right]}\right\}}^{2}},\;\tilde{\xi }=\frac{\left(\xi -\xi_{e}\right)}{\xi_{e}}.$$

The change in the work of critical cluster formation caused by deviations in the state of the liquid from metastable equilibrium is described by the factor Θ being a function of the structural order parameter, ξ, and its equilibrium value, $\xi_{e}$ (see Figure 1). It is equal to one for $\xi =\xi_{e}$.

Finally, $J_{0}$ reflects the kinetic mechanism of cluster formation and growth; in the present analysis, it is chosen as

(35) $$J_{0}=c\sqrt{\frac{\sigma }{k_{B}T}}\left(\frac{2D}{d_{0}}\right).$$

Here, *D* is the diffusion coefficient governing the aggregation kinetics. We suppose that the kinetic mechanism of aggregation is the same for both nucleation and growth and is governed by a diffusion coefficient, *D*, which can be written, in general, as

(36) $$D=D_{0}\exp\left(-\frac{E_{D}}{k_{B}T}\right).$$

The activation energy for diffusion, $E_{D}=E_{D}\left(T\right)$, depends on temperature, pressure, and composition. For the macroscopic linear growth rate, *u*, we use the commonly employed relation

(37) $$u=\frac{D}{4d_{0}}\left[1-\exp\left(-\frac{\Delta \mu }{k_{B}T}\right)\right]$$

setting $\omega_{n}=1$.

In the numerical computations, we express the diffusion coefficient for the description of both nucleation and crystal growth as

(38) $$D=\left\{\begin{array}{cc} \left(\frac{d_{0}^{2}}{\tau_{0}}\right)\exp\left(-7.5\frac{T_{g}}{T-T_{0}}\right) & \mathrm{for}\;T\geq T_{d}\\ \; & \\ \left(\frac{d_{0}^{2}}{\tau_{0}}\right)\exp\left(-7.5\frac{T_{g}}{T}\left(\frac{T_{d}}{T_{d}-T_{0}}\right)\right) & \mathrm{for}\;T\leq T_{d} \end{array}\right.$$

with

(39) $$\tau_{0}=\frac{h}{k_{B}T},\;T_{0}=\frac{T_{m}}{2}.$$

**Figure 2:**
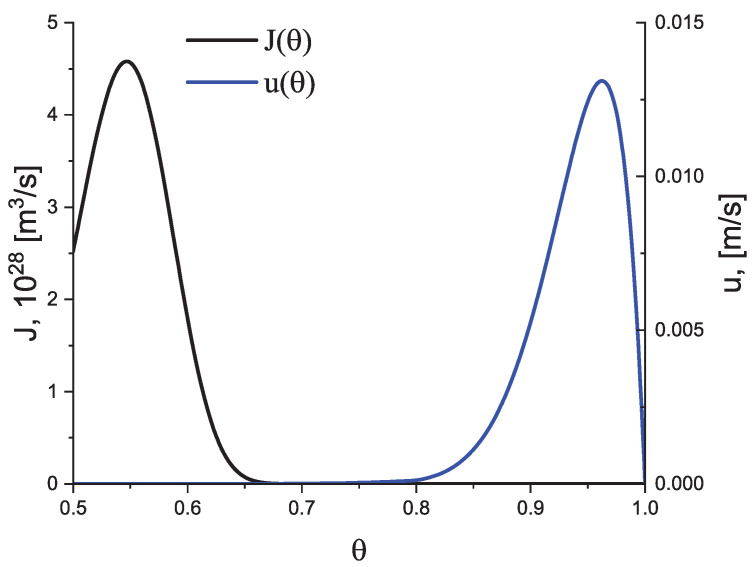
Dependence of the steady-state nucleation rate, *J*, and the growth rate, *u*, on temperature in reduced coordinates, $\theta =T/T_{m}$, for the model employed here in the computations in the limiting case that the liquid is always in a metastable state.

Here, *h* is Planck’s constant and $T_{d}$ is the temperature of the decoupling of diffusion and viscosity (relaxation), i.e., the temperature at which the Stokes–Einstein–Eyring equation breaks down. We set it equal to $T_{d}=1.2T_{g}$, where $T_{g}$ is the glass transition temperature according to the definition by Tammann (see, e.g., [5,6,38]) correlating it with a Newtonian viscosity, η, equal to $\eta \left(T\right)≅10^{12}$ Pa s at $T=T_{g}$. In the absence of deviations of the liquid from metastable equilibrium, the dependence of the steady-state nucleation and growth rates on temperature have a form in this model approach as illustrated in Figure 2.

The change in the structural order parameter with time is given by

(40) $$\frac{d\xi }{dt}=-\frac{1}{\tau_{R}\left(T,p,\xi \right)}\left(\xi -\xi_{e}\right).$$

In the computations, we employ the relation

(41) $$\tau_{R}=\tau_{0}\exp\left(7.5\frac{T_{g}}{T-T_{0}}\right).$$

Below $T_{d}$, more complex relaxation laws may have to be utilized for a quantitatively correct description of relaxation [5,6,22,39,40]. As a rule, they enhance the effective relaxation times. The basic qualitative conclusions of our analysis are not affected by the approximation employed in our study.

Assuming a certain (constant) rate of change in temperature,

(42) $$q=\frac{dT}{dt},$$

with different signs of the parameter *q* (q<0 for cooling and q>0 for heating processes); Equation (40) then takes the form

(43) $$\frac{d\xi }{dT}=-\frac{1}{q\tau_{R}}\left(\xi -\xi_{e}\right).$$

Solving this equation, we can determine ξ(T) for any desired temperature below the melting temperature, $T_{m}$, of the substance under consideration.

The typical course of the ξ(T)–curves in cooling and heating is shown in Figure 1b. Note that $\ln\xi_{e}<0$ always holds (cf. Equation (29) and Figure 1a) and, while $\tilde{\xi }$ is approaching zero in the course of an isothermal relaxation process, the parameter Θ in Equations (33) and (34) tends to one. Deviations in the state of the liquid from metastable equilibrium may lead to both an increase (for $\xi >\xi_{e}$) and a decrease (for $\xi <\xi_{e}$) in the surface tension. In contrast, deviations from metastable equilibrium always result in an increase in the thermodynamic driving force. It follows that, in isothermal relaxation, Θ may approach the final value from above (for $\xi >\xi_{e}$ leading to Θ≥1) or from below (for $\xi <\xi_{e}$ leading to Θ≤1).

### 2.3. Possible Extensions

For a quantitatively more correct analysis, more general relations may have to be used. In particular, (i) in cooling and heating, both thermal and athermal nucleation have to be taken into consideration having the following meaning. In classical nucleation theory, the term nucleation rate is usually identified with the rate of the formation of critical clusters. For constant external and internal conditions, this rate is at the same time equal to the change in the total number of clusters exceeding the critical size, $j_{c}$.

However, if the state of the system is changed in the course of the transformation, either due to a variation in the external conditions or internal processes (decrease in supersaturation), then the rate of the formation of critical clusters is not equal to the rate of change in the total number of clusters exceeding the critical size. Indeed, if we introduce the notations

(44) $$N\left(j\geq j_{c},t\right)=\int_{j_{c}}^{\infty }N\left(j,t\right)\;dj,$$

where $N(j\geq j_{c},t)$ is the number of clusters with sizes $j\geq j_{c}$ at time *t* in the system, and

(45) $$J\left(j\geq j_{c},t\right)=\frac{dN(t)}{dt}$$

for their rate of formation, then

(46) $$J=\frac{\partial }{\partial t}\int_{j_{c}}^{\infty }N\left(j,t\right)\;dj$$

holds.

With the continuity equation connecting flux in cluster size space and the number of supercritical clusters (c.f. [5,6]), for time-independent supersaturations $\left(j_{c}=\mathrm{constant}\right)$, one obtains

(47) $$J\left(j\geq j_{c},t\right)=J\left(j_{c},t\right),$$

while for time-dependent situations

(48) $$J\left(j\geq j_{c},t\right)=J\left(j_{c},t\right)-N\left(j_{c},t\right)\frac{\partial j_{c}}{\partial t}$$

is obtained. The first term in Equation (48) describes the stochastic process of the formation of supercritical clusters connected with thermal fluctuations in the system. The second term accounts for the process of athermal nucleation, which is a consequence of the change in the critical cluster size caused here by variations in temperature. In cooling, the critical cluster size decreases with decreasing temperature, giving an additional positive contribution to the total number of clusters; in heating, we have the opposite situation.

In order to employ the above equation, we have to specify the values of the critical cluster size, $j_{c}$, and the value of the number of critical clusters per unit volume, $N(j_{c},t)$, at some given time, *t*. The latter parameter can be determined via classical nucleation theory. In order to have an estimate for $N(j_{c},t)$, we can use the expression for the steady-state cluster size distribution [5,6]

(49) $$N\left(j_{c},t\right)=\frac{N(1)}{2}\exp\left(-\frac{W_{c}}{k_{B}T}\right),$$

where N(1) is the number of monomers per unit volume in the system and $W_{c}$ the actual value of the work of critical cluster formation. This approach implies that the change in the external parameters is not too fast so that steady-state conditions can be established in the range up to the critical cluster size at each moment of time. Combining the above given relations and Equations (10) and (11), we obtain

(50) $$\alpha_{n}\left(t\right)=1-\exp\left(-Y_{n}\left(t\right)\right),$$

(51) $$Y_{n}\left(t\right)=\omega_{n}\int_{0}^{t}\left\{J\left(t^{\prime }\right)-\frac{N(1)}{2}\exp\left(-\frac{W_{c}\left(t^{\prime }\right)}{k_{B}T}\right)\frac{\partial j_{c}\left(t^{\prime }\right)}{\partial t^{\prime }}\right\}dt^{\prime }{\left(\int_{t^{\prime }}^{t}u\left(t^{\prime \prime }\right)dt^{\prime \prime }\right)}^{n},$$

or, with Equation (27),

(52) $$Y_{n}\left(t\right)=\omega_{n}\int_{0}^{t}\left\{J\left(t^{\prime }\right)\left[1-\frac{N(1)}{2J_{0}}\frac{\partial j_{c}\left(t^{\prime }\right)}{\partial t^{\prime }}\right]\right\}dt^{\prime }{\left(\int_{t^{\prime }}^{t}u\left(t^{\prime \prime }\right)dt^{\prime \prime }\right)}^{n}.$$

The account of athermal nucleation leads, consequently, to a change in the prefactor, $J_{0}$, in the expression for the steady-state nucleation rate (cf. Equation (27)).

As a second possible generalization, (ii) time-lag effects in nucleation have to be accounted for [5,6,34,41,42,43,44]. Its incorporation requires an appropriate description of the approach to steady-state conditions and estimates of the time lag in nucleation, $\tau_{ns}$. The time lag in nucleation is the time required to reach steady-state cluster size distributions [5,6,45]. Assuming that initially the liquid consists only of monomers (atoms, molecules), the time lag in nucleation can be estimated as [5,6,21]

(53) $$\tau_{ns}=\frac{\omega }{2}\left(\frac{k_{B}T}{\sigma d_{0}^{2}}\right)\left(\frac{R_{c}^{2}}{D\tau_{R}}\right)\tau_{R}.$$

The numerical factor ω varies in the range 1≤ω≤4 depending on the method employed in the derivation of Equation (53). Above the decoupling temperature, $T_{d}$, the product $D\tau_{R}$ becomes widely independent of temperature since in this range the Stokes–Einstein–Eyring relation may be employed, below $T_{d}$ it does not. However, considering cooling and heating, the value of the time lag depends also on prehistory [42,43]; it will be smaller in general as compared to the estimate given by this relation. Time-lag effects can for sure be neglected if the characteristic time of change in the temperature, $\tau_{T}$, in cooling and heating is small as compared with the time lag in nucleation, i.e., if the condition

(54) $$\tau_{ns}\ll \tau_{T}=\frac{T}{\left|dT/dt\right|}$$

holds.

In general, the average time of the formation of the first supercritical nucleus can be expressed in a good approximation as [41]

(55) $$\langle \tau \rangle ≅\tau_{ns}+{\langle \tau \rangle }_{ss},\;{\langle \tau \rangle }_{ss}=\frac{1}{J_{st}V}.$$

Provided steady-state conditions with respect to nucleation are established, the following relation,

(56) $$\langle \tau \rangle ={\langle \tau \rangle }_{ss}=\frac{1}{J_{st}V},$$

holds for the time required to form the first supercritical cluster under such conditions. The condition $\langle \tau \rangle \ll \tau_{R}$ is fulfilled in the temperature range near to the glass transition temperature and below. In this temperature range, moreover, $\langle \tau \rangle ≅\tau_{ns}$ is a good approximation. The effects of relaxation of the liquid on the overall crystallization can be expected, theoretically, to occur at $T\leq T_{g}$. They then also affect the overall crystallization kinetics at higher temperatures.

In a third direction of advancement of the theoretical approach, (iii) the effect of the interplay of elastic stress evolution and stress relaxation on nucleation and growth [5,6,28,29,30,31,32] in the temperature range near and below the glass transition temperature has to be accounted for. For crystal nucleation in viscous liquids, the effective value of the stress parameter ε, the elastic energy per particle of the crystal phase, is determined by the interplay of stress evolution (caused by the formation of a crystallite) and stress relaxation accompanying this process. Assuming, as carried out here, that relaxation is described by Maxwell’s relaxation law, the effective value of ε for a crystallite of critical size is given by

(57) $$\frac{\varepsilon (j_{c})}{\varepsilon_{0}}≅\frac{\tau_{R}}{\tau_{ns}}\left[1-\exp\left(-\frac{\tau_{ns}}{\tau_{R}}\right)\right].$$

Here, $\varepsilon_{0}$ is the respective value for crystallization in a Hookean solid. More general relations accounting for more complex relaxation laws are given in [5,6,28,29,30,31,32]. They lead to qualitatively similar results. The effect of the interplay of the evolution of elastic stresses caused by crystal nucleation and relaxation in the description of nucleation is determined by the ratio $\tau_{R}/\tau_{ns}$. It also results in an increase in the surface tension as described in [21]. The effect of the interplay of stress evolution and relaxation in crystal growth in glass-forming liquids is described in [46,47]; it is also determined by this ratio of characteristic timescales, $\tau_{R}/\tau_{ns}$.

Further extensions of the approach described above will eventually be required to account for (iv) the possible dependence of the bulk properties of critical clusters on supersaturation [37,48], (v) self-consistency corrections of the steady-state nucleation rate [49], (vi) the possible dependence of the kinetic coefficients on the structural order parameter [5,6], (vii) a more precise description of the scenario and the kinetics of relaxation (see e.g., [5,6,21,28,34]), (viii) a more detailed description of the glass-forming melts including the extension of the model to several structural order parameters, (ix) the account of Ostwald’s rule of stages (or Ostwald’s step rule) [50], of secondary nucleation [51] leading also to modifications of the growth rate as compared to Equation (37), of both bulk and surface crystallization [10,52], and of the evolution of rigid amorphous fractions [53] on the overall crystallization process. Another circle of questions is connected with (x) the problem as to whether a spinodal or a pseudo-spinodal may exist and, consequently, affect melt crystallization [43,54,55,56,57]. The latter question is addressed in detail in Appendix A.

From our point of view, all of these (and possible further) factors are expected to eventually affect, quantitatively, the results of our analysis but not the general qualitative features of the effect of deviations of the liquid from metastable equilibrium and its relaxation to this state on overall crystallization as described in the next sections.

## 3. Results of Numerical Computations

**Figure 3:**
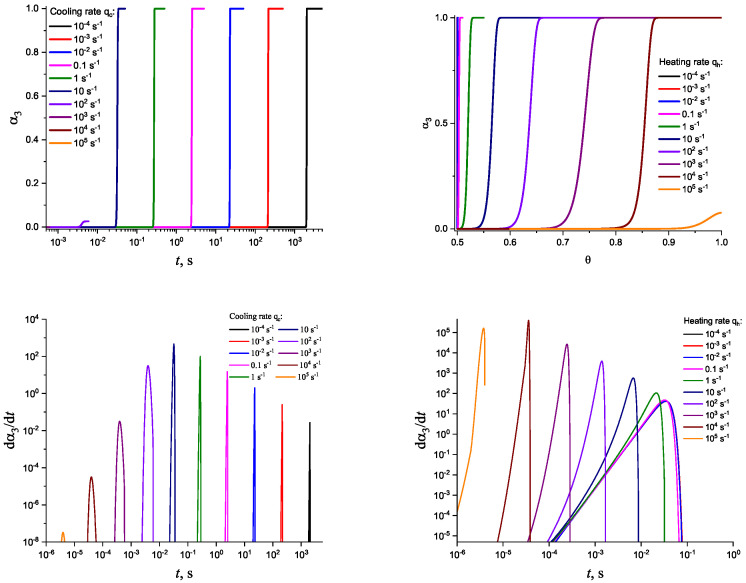
Determination of the degree, $\alpha_{3}$, and the rate, $d\alpha_{3}/dt$, of overall crystallization in dependence on time, *t*, in cooling (**left side**) and heating (**right side**) for sets of cooling ($q_{c}$) and heating ($q_{h}$) rates as shown in the figures. In the presentation of the results of the numerical computations, we always express the heating rate in reduced variables as $q=d(T/T_{m})/dt$. Cooling is started at a temperature equal to $T=T_{m}$, while heating is supposed to start at a temperature $T=(T_{m}/2)$.

**Figure 4:**
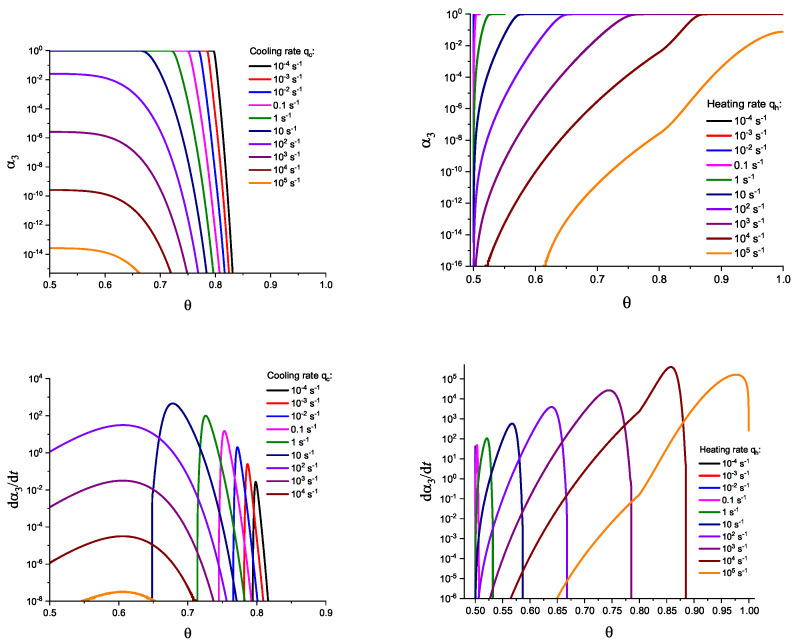
Determination of the degree, $\alpha_{3}$, and the rate, $d\alpha_{3}/dt$, of overall crystallization in dependence on temperature, $\theta =T/T_{m}$, in cooling (**left side**) and heating (**right side**) for sets of cooling ($q_{c}$) and heating ($q_{h}$) rates as shown in the figures.

In Figure 3 and Figure 4, we present the results of numerical computations modeling the evolution of the degree of overall crystallization, $\alpha_{n}$, for the model considered assuming steady-state nucleation and growth in three (n=3) independent directions. In the computations shown in these figures, it is assumed that deviations in the state of the liquid from metastable equilibrium can be neglected. The steady-state nucleation rate and the growth rates are then described by a dependence on temperature as shown in Figure 2. In cooling, the process is started at the melting temperature, and in heating, at sufficiently low temperatures equal to $T=T_{m}/2$. In the initial states, the liquid is considered to be free of crystalline particles. In Figure 3, the process is shown in terms of dependence on temperature, while in Figure 4, the same quantities are given as functions of the reduced temperature, $\theta =T/T_{m}$. A significant dependence of the shapes of the respective curves on cooling and heating rates is observed. In particular, the crystallization peak temperatures (corresponding to $d^{2}\alpha_{n}/dt^{2}=0$ or $d^{2}\alpha_{n}/d\theta^{2}=0$) are shifted to lower temperatures with increasing values of the cooling rate, $q_{c}$. In contrast, for heating processes, the crystallization peak temperature is shifted to higher values with increasing heating rates, $q_{h}$.

**Figure A1:**
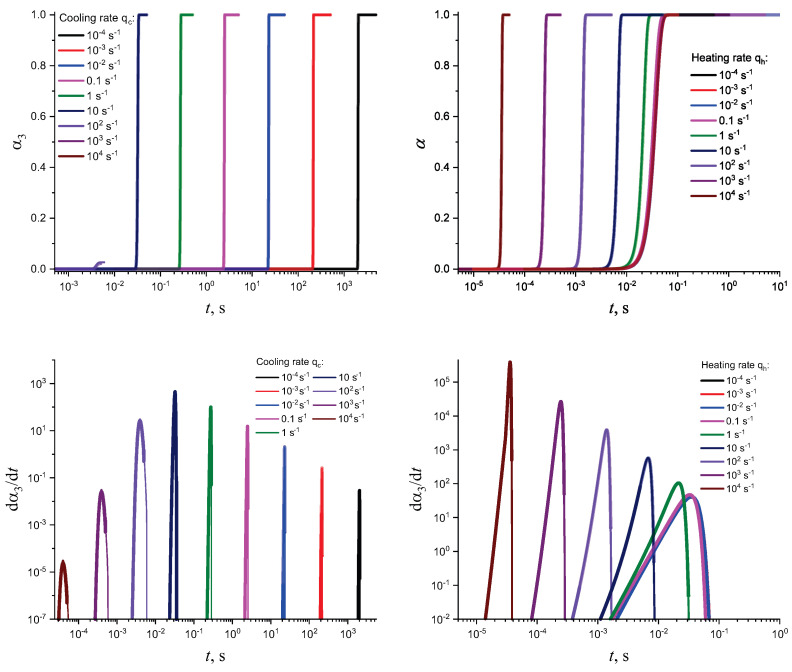
Determination of the degree, $\alpha_{3}$, and the rate, $d\alpha_{3}/dt$, of overall crystallization in dependence on time, *t*, in cooling (**left side**) and heating (**right side**) for sets of cooling ($q_{c}$) and heating ($q_{h}$) rates as shown in the figures. In contrast to Figure 3, here the effect of deviations in the state of the liquid from metastable equilibrium is accounted for. The computations are performed utilizing Equations (32) and (33).

**Figure A2:**
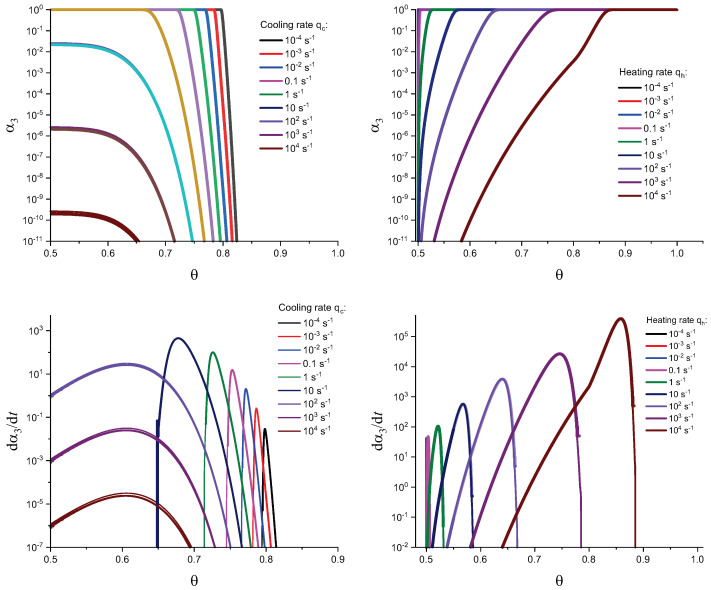
Determination of the degree, $\alpha_{3}$, and the rate, $d\alpha_{3}/dt$, of overall crystallization in dependence on temperature, $\theta =T/T_{m}$, in cooling (left side) and heating (right side) for sets of cooling ($q_{c}$) and heating ($q_{h}$) rates as shown in the figures. In contrast to Figure 4, here the effect of deviations in the state of the liquid from metastable equilibrium is accounted for. The computations are performed utilizing Equations (32) and (33).

For the next step, we again present the results of numerical computations modeling the evolution of the degree of overall crystallization, $\alpha_{n}$, for the model considered assuming steady-state nucleation and growth in three (n=3) independent directions. However, in the computations shown in these figures, it is now assumed that deviations in the state of the liquid from metastable equilibrium have to be accounted for. Deviations of the thermodynamic driving force and the surface tension caused by deviations from metastable equilibrium are described in a first approach by Equations (32) and (33). A comparison with Figure 3 and Figure 4 shows that deviations in the curves occur but they are relatively small. For comparison, we present them anyway in Appendix B (in Figure A1 and Figure A2).

In addition, we employ here an alternative model: an independent approach for the determination of the effect of deviations in the thermodynamic driving force and surface tension caused by deviations from metastable equilibrium as described in detail in [37]. In this approach, we determine these quantities via the relations

(58) $$\Delta g\left(T,p;\xi \right)≅\Delta h_{m}\left(1-\frac{T}{T_{m}}\right)\left[1-\frac{\Delta c_{p}\left(T_{m},p_{m}\right)}{2\Delta s_{m}}\left(1-\frac{T}{T_{m}}\right)+\Omega_{\Delta g}{\tilde{\xi }}^{2}\right]$$

and

(59) $$\frac{\sigma (T,p,\xi )}{\sigma (T_{m},p_{m})}=\frac{T}{T_{m}}\left[1-\frac{\Delta c_{p}}{\Delta s_{m}}\left(1-\frac{T}{T_{m}}\right)+\Omega_{\sigma }\tilde{\xi }\right]\;.$$

**Figure 5:**
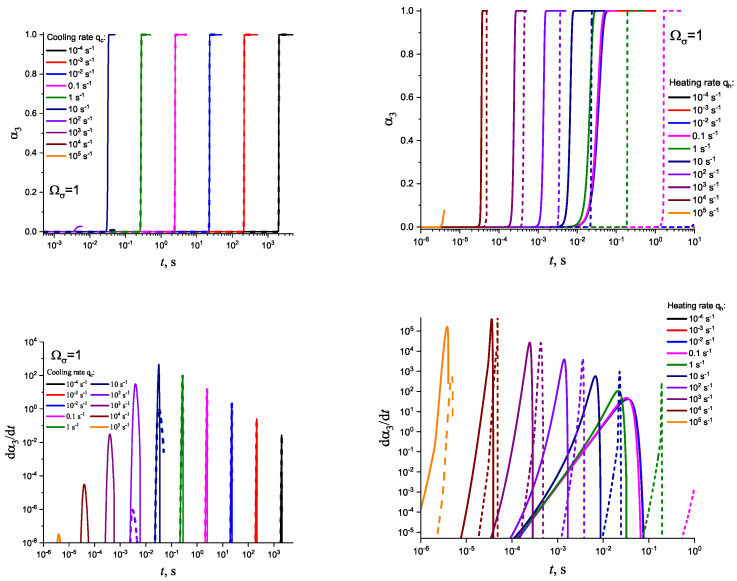
Determination of the degree, $\alpha_{3}$, and the rate, $d\alpha_{3}/dt$, of overall crystallization in dependence on time, *t*, in cooling (**left side**) and heating (**right side**) for sets of cooling ($q_{c}$) and heating ($q_{h}$) rates as shown in the figures. In contrast to Figure 3, here the effect of deviations in the state of the liquid from metastable equilibrium is accounted for. The computations are performed utilizing Equations (58) and (59) with $\Omega_{\Delta g}=1$ and $\Omega_{\sigma }=1$. The results obtained in such a way are shown by dashed curves; the curves shown in Figure 3 and Figure 4 are given for comparison as full curves again.

**Figure 6:**
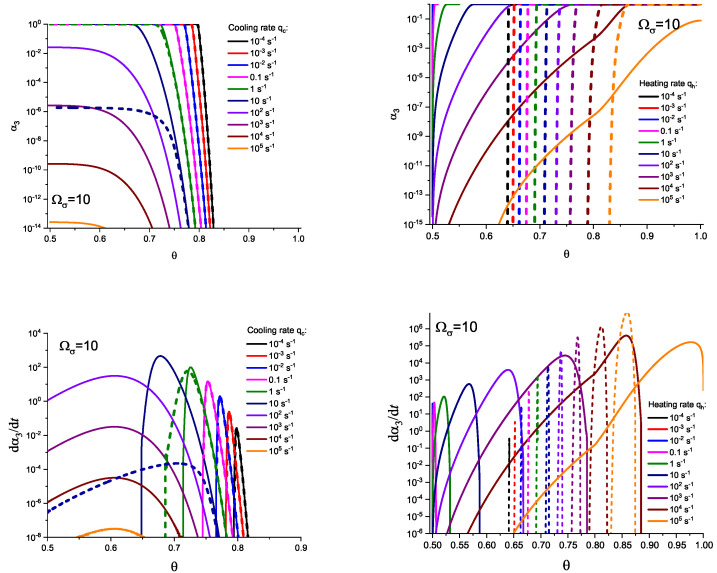
Determination of the degree, $\alpha_{3}$, and the rate, $d\alpha_{3}/dt$, of overall crystallization in dependence on temperature, $\theta =T/T_{m}$, in cooling (**left side**) and heating (**right side**) for sets of cooling ($q_{c}$) and heating ($q_{h}$) rates as shown in the figures. In contrast to Figure 4, here the effect of deviations in the state of the liquid from metastable equilibrium is accounted for. The computations are performed utilizing Equations (58) and (59) with $\Omega_{\Delta g}=1$ and $\Omega_{\sigma }=10$. The results obtained in such a way are shown by dashed curves; the curves shown in Figure 3 and Figure 4 are given for comparison as full curves again.

**Figure 7:**
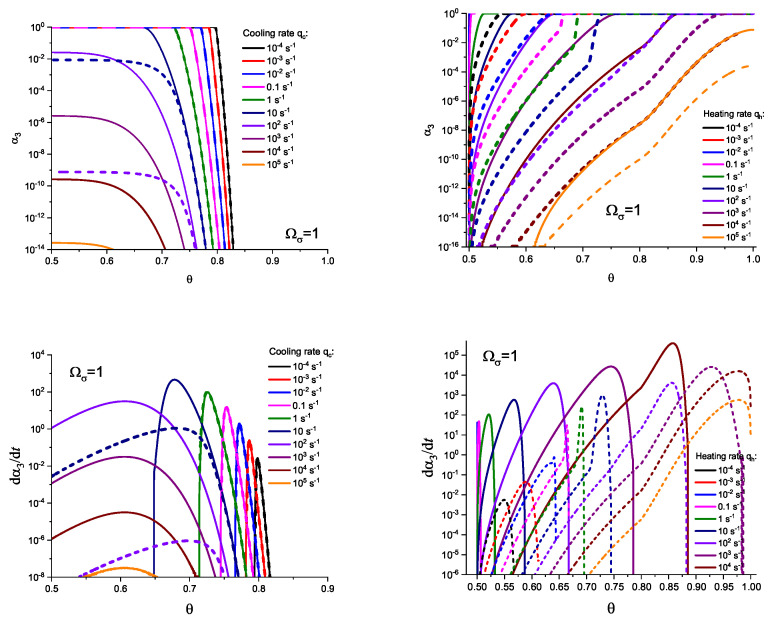
Determination of the degree, $\alpha_{3}$, and the rate, $d\alpha_{3}/dt$, of overall crystallization in dependence on temperature, $\theta =T/T_{m}$, in cooling (**left side**) and heating (**right side**) for sets of cooling ($q_{c}$) and heating ($q_{h}$) rates as shown in the figures. In contrast to Figure 4, here the effect of deviations in the state of the liquid from metastable equilibrium is accounted for. The computations are performed utilizing Equations (58) and (59) with $\Omega_{\Delta g}=1$ and $\Omega_{\sigma }=1$. The results obtained in such a way are shown by dashed curves; the curves shown in Figure 3 and Figure 4 are given for comparison as full curves again.

**Figure 8:**
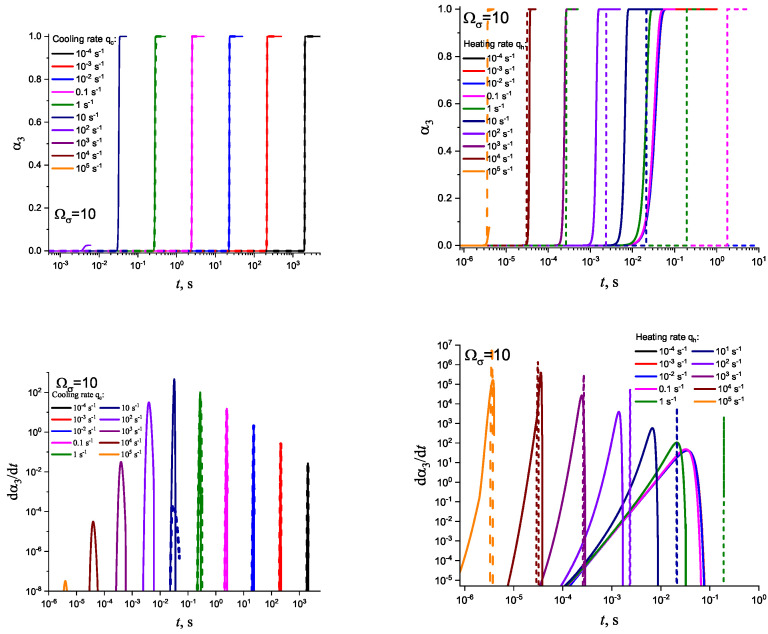
Determination of the degree, $\alpha_{3}$, and the rate, $d\alpha_{3}/dt$, of overall crystallization in dependence on time, *t*, in cooling (**left side**) and heating (**right side**) for sets of cooling ($q_{c}$) and heating ($q_{h}$) rates as shown in the figures. In contrast to Figure 3, here the effect of deviations in the state of the liquid from metastable equilibrium is accounted for. The computations are performed utilizing Equations (58) and (59) with $\Omega_{\Delta g}=1$ and $\Omega_{\sigma }=10$. The results obtained in such a way are shown by dashed curves; the curves shown in Figure 3 and Figure 4 are given for comparison as full curves again.

**Figure 9:**
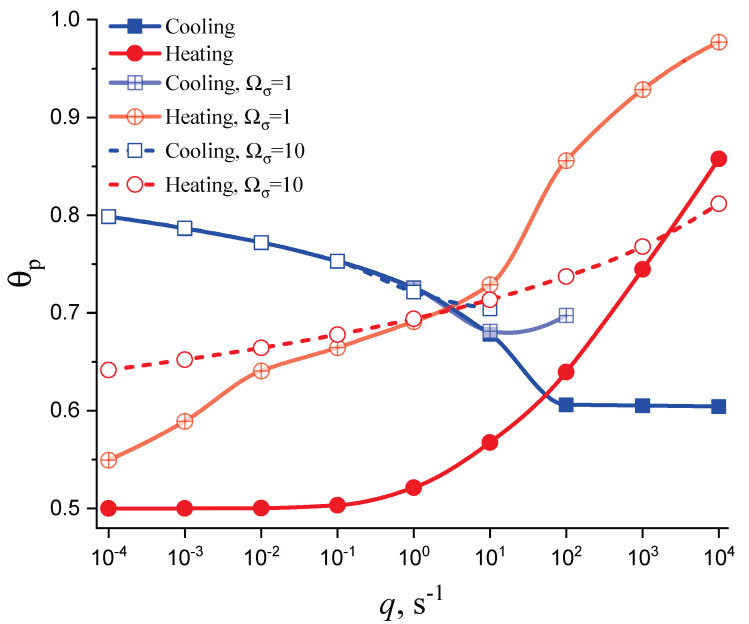
Crystallization peak temperatures, $\theta_{p}=T_{p}/T_{m}$, in dependence on cooling (blue) and heating (red) rates as obtained from the numerical computations. The results are given for nucleation and growth in metastable liquids (presented in Figure 3 and Figure 4) and accounting for deviations from metastability in the form as shown in Figure 5 and Figure 7 ($\Omega_{\sigma }=1$), respectively, and Figure 6 and Figure 8 ($\Omega_{\sigma }=10$).

Different models are characterized here by different values of the parameters $\Omega_{\Delta g}$ and $\Omega_{\sigma }$. The results of computations shown in Figure 5 and Figure 6 are obtained setting $\Omega_{\Delta g}=1$ and $\Omega_{\sigma }$ equal to two different values, $\Omega_{\sigma }=1$ (Figure 5 and Figure 7) and $\Omega_{\sigma }=10$ (Figure 6 and Figure 8), correspondingly. The results obtained in such a way are shown by dashed curves; the curves shown in Figure 3 and Figure 4 are given for comparison as full curves again. The dependence of the crystallization peak temperatures on the cooling and heating rates obtained numerically for the different cases discussed is presented in Figure 9.

The main conclusions can be summarized as follows: (i) Deviations in the liquid from metastable equilibrium may significantly affect the overall course of the crystallization kinetics. (ii) The influence of these deviations is more important for heating as compared to cooling processes. (iii) Both in cooling and heating, the overall crystallization process may not be completed for sufficiently high cooling and heating rates in agreement with the experiment. This effect determines to a large degree the glass-forming ability of a given substance as discussed in connection with the kinetic criteria of glass formation in terms of the TTT diagrams [5,6,58] utilizing the JMAK equations. It is also found in the computations that the critical heating rate is about one order of magnitude higher than the critical cooling rate, as observed commonly in experiments [59,60,61,62]. (iv) In heating, deviations from metastable equilibrium originally inhibit nucleation (as far as the condition $\tilde{\xi }\geq 0$ holds). This effect occurs for relatively low temperatures (cf. Figure 1). For higher temperatures (at $\tilde{\xi }\leq 0$), we have the opposite situation. Deviations from equilibrium accelerate nucleation. The mentioned effects increase with an increase in the value of the parameter $\Omega_{\sigma }$ determining the magnitude of changes in the surface tension caused by deviations from metastable equilibrium. (v) The nucleation peak temperatures depend significantly on the cooling and heating rates. The peak temperatures in cooling are weakly affected by the degree of deviation of the liquid from equilibrium; in heating, the effect is large. These dependencies and the origin of the mentioned differences are studied theoretically in detail in the subsequent section (Section 4).

## 4. Theoretical Analysis

### 4.1. Some General Considerations

In the preceding section, the results of numerical computations are presented showing the change in the degree of crystallization of a liquid in cooling and heating modeled in terms of the JMAK equation, Equations (10) and (11),

(60) $$\alpha_{n}\left(t\right)=1-\exp\left(-Y_{n}\left(t\right)\right),\;Y_{n}\left(t\right)=\omega_{n}\int_{0}^{t}J\left(t^{\prime }\right)dt^{\prime }{\left(\int_{t^{\prime }}^{t}u\left(t^{\prime \prime }\right)dt^{\prime \prime }\right)}^{n}.$$

The main attention is devoted, hereby, to the analysis of the problem with regards to under which conditions and to what extent is crystallization affected by the relaxation of the glass-forming liquid to the respective metastable equilibrium state. As discussed in detail in previous papers [21,23,37,43], it is determined by the ratio of the average time of formation of the first supercritical nucleus, 〈τ〉, and the relaxation time, $\tau_{R}$, being equal to the timescale in the approach to thermodynamic equilibrium. Deviations from metastable equilibrium and relaxation processes to it may affect crystal nucleation and growth if the condition $\langle \tau \rangle \ll \tau_{R}$ is fulfilled. In cooling and heating, the interplay of relaxation and nucleation and growth significantly affects the whole course of overall crystallization; in particular, the dependence of the crystallization peak temperatures on cooling and heating rates. These dependencies are shown in Figure 9 as obtained via the numerical computations. Section 4.2 is devoted to an analytical description of these results.

### 4.2. Cold Crystallization Peak Temperature in Heating as a Function of the Heating Rate: Homogeneous Nucleation

Solving the above given relations numerically, we can determine, as a special case, the temperatures of the crystallization peaks, $T_{p}$, widely discussed in the analysis of the experimental data in cooling and heating (e.g., [1,2,3,17,18,19,20,63,64]). In the present section, we derive simple relations for the dependence of the cold crystallization peak temperature on the heating rate, and, similarly, for the crystallization peak temperature in cooling for the case of heterogeneous nucleation, extending the analysis performed in [63]. In this procedure, certain approximations are employed connected with the locations of the maxima of nucleation and growth rates in terms of dependence on temperature.

**Figure 10:**
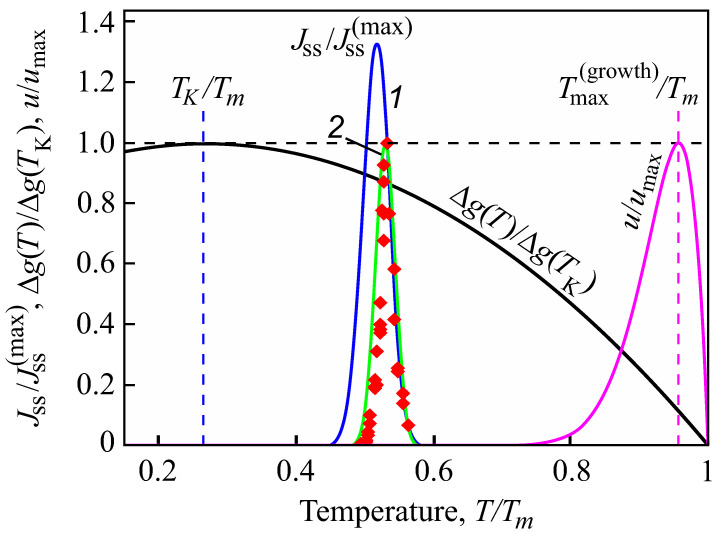
Normalized steady-state nucleation rate, $J_{ss}/J_{ss}^{\left(max\right)}$, and normalized crystal growth rate, $u/u_{max}$, in dependence on reduced temperature, $T/T_{m}$. Here $J_{ss}^{\left(max\right)}$ is the maximum nucleation rate and $u_{max}$ is the maximum growth rate obtained via experiment; $T_{m}$ is the melting or liquidus temperature. The blue curve (1) shows the theoretical result when the kinetic term in the expression for the nucleation rate is determined via appropriate diffusion coefficients; the green curve (2) is drawn under the assumption of validity of the Stokes–Einstein–Eyring equation, allowing one to replace the diffusion coefficient with viscosity. Its wide coincidence with experimental data is reached by employing appropriate expressions for the curvature dependence of the surface tension (for details, see [43]). The reduced thermodynamic driving force, $\Delta g\left(T\right)/\Delta g\left(T_{K}\right)$, is also shown; it has a maximum at the Kauzmann temperature, $T_{K}$ [68]. It is evident that crystallization occurs only in a relatively small temperature range. Typically, the maximum of the growth rate, $T_{max}^{\left(growth\right)}$, is located at temperatures much higher than the maximum of the steady-state nucleation nucleation rate [65,66,67], as shown here in the figure. The figure is taken from [56] (Creative Commons Attribution License).

Indeed, from a theoretical point of view, it can be shown that intensive homogeneous nucleation occurs in a relatively small range, ΔT, of temperatures (see Figure 10), with a maximum near to the glass transition temperature, $T_{g}$, defined in the classical form proposed by Tammann [38] as related to a viscosity of $10^{12}$ Pa s. This maximum is caused by the interplay of thermodynamic and kinetic factors dominating the crystal nucleation process. The kinetic factor is correlated with the diffusion coefficients significantly decreasing with decreasing temperature. The maximum of the growth rates is located, as a rule, at much higher temperatures [65,66,67].

For this reason, in experiments, Tammann’s development method (see e.g., [5,6,59,60,61,62,65]) is commonly employed in the analysis of crystal nucleation. The formation of crystal nuclei is stimulated by choosing some well-defined nucleation temperatures; then, in order to detect these nuclei, the temperature is switched to higher values to allow them to grow to detectable experimental sizes over reasonable experimental timescales. However, for heterogeneous nucleation, the maximum of the nucleation rate may be located at higher temperatures compared to the maximum of the growth rate quite near to the melting temperature [66,67]. These differences in the locations of the maxima of nucleation and growth rates supply us, as will be shown, with the key to understanding the differences in the overall crystallization behavior in cooling and heating observed experimentally and reflected also in the numerical computations.

Considering first crystallization in heating with a constant rate, q=dT/dt, Equations (60) may be reformulated as

(61) $$\alpha_{n}\left(T\right)=1-\exp\left[-Y_{n}\left(T\right)\right],\;Y_{n}\left(T\right)=\frac{\omega_{n}}{q^{n+1}}\int_{0}^{T}J\left(T^{\prime }\right)dT^{\prime }{\left(\int_{T^{\prime }}^{T}u\left(T^{\prime \prime }\right)dT^{\prime \prime }\right)}^{n}.$$

We can then approximately describe the number of supercritical crystallites, *N*, formed via homogeneous nucleation in heating, by the relation

(62) $$N\left(q\right)=\int_{0}^{t}J\left(t^{\prime }\right)dt^{\prime }≅\frac{1}{q}J\left(T_{max}^{\left(nucl\right)}\right)\Delta T^{\left(nucl\right)}.$$

Here, $T_{max}^{\left(nucl\right)}$ is the temperature corresponding to the maximum of the steady-state nucleation rate, and $\Delta T^{\left(nucl\right)}$ the temperature range, where nucleation effectively occurs. It can be determined by

(63) $$\Delta T^{\left(nucl\right)}≅\int_{0}^{T_{m}}\frac{J(T)}{J(T_{max}^{\left(nucl\right)})}dT.$$

Increasing the heating rate, consequently, results in a decrease in the number of supercritical clusters formed in a given heating run.

Utilizing these relations, Equation (61) can be reformulated as

(64) $$\alpha_{n}\left(T\right)=1-\exp\left[-Y_{n}\left(T\right)\right],\;Y_{n}\left(T\right)=\frac{\omega_{n}}{q^{n+1}}J\left(T_{max}^{\left(nucl\right)}\right)\Delta T^{\left(nucl\right)}{\left(\int_{T_{max}^{\left(nucl\right)}}^{T}u\left(T^{\prime \prime }\right)dT^{\prime \prime }\right)}^{n}.$$

Accounting for the mathematical identity

(65) $$\int_{a}^{b}y\left(x\right)dx=y\left(\langle x\rangle \right)\left(b-a\right),\;a\leq \langle x\rangle \leq b,$$

the second term in this relation can be rewritten as

(66) $$Y_{n}\left(T\right)=\frac{\omega_{n}}{q^{n+1}}J\left(T_{max}^{\left(nucl\right)}\right)\Delta T^{\left(nucl\right)}{\left(u\left(\langle T\rangle \right)\left(T-T_{max}^{\left(nucl\right)}\right)\right)}^{n}.$$

Here, 〈T〉 obeys the condition $T_{max}^{nucl}\leq \langle T\rangle \leq T$. Setting 〈T〉 in u(〈T〉) approximately equal to $\langle T\rangle =T_{max}^{\left(nucl\right)}+\left(1/2\right)\left(T-T_{max}^{\left(nucl\right)}\right)$, we obtain in this way a simple first estimate for the dependence of the change in the overall crystallization on temperature. However, here we concentrate the attention on one particular feature of the $\alpha_{n}\left(T\right)$–curves, the dependence of the crystallization peak temperature, $T_{p}$, on the heating rate, *q*.

The cold crystallization peak in heating corresponds to the maximum of the derivatives, $\left(d\alpha_{n}/dT\right)$, of the overall crystallization curves or, equivalently, the inflexion point of the $\alpha_{n}\left(T\right)$–curves. It is determined by $(d^{2}\alpha_{n}\left(T\right)/dT^{2})=0$. Utilizing Equation (64), we obtain as a consequence

(67) $${\left(\frac{dY_{n}\left(T\right)}{dT}\right)}^{2}-\frac{d}{dT}\left(\frac{dY_{n}\left(T\right)}{dT}\right)=0.$$

This is the most general relation for the determination of the crystallization peak temperature. However, its application leads to quite complex expressions. On the other hand, Equation (67) implies that

(68) $$Y_{n}\left(T_{p},q\right)=\mathrm{constant}$$

is fulfilled.

The latter condition is realized if

(69) $${\tilde{Y}}_{n}\left(T_{p},q\right)=\frac{1}{q^{\left(n+1\right)}}{\left(\int_{T_{max}^{\left(nucl\right)}}^{T_{p}}u\left(T\right)dT\right)}^{n}=\mathrm{constant}.$$

Taking the differential of ${\tilde{Y}}_{n}\left(T_{p},q\right)$, we obtain

(70) $$\frac{d{\tilde{Y}}_{n}\left(T_{p},q\right)}{dT_{p}}dT_{p}+\frac{d{\tilde{Y}}_{n}\left(T_{p},q\right)}{dq}dq=0$$

and, computing the derivatives,

(71) $$\frac{dT_{p}}{dq}=\frac{1}{q}\left(\left(\frac{n+1}{n}\right)\frac{1}{u(T_{p})}\int_{T_{max}^{\left(nucl\right)}}^{T_{p}}u\left(T\right)dT\right).$$

Employing Equation (65), we may rewrite this relation as

(72) $$\frac{dT_{p}}{dq}=\frac{1}{q}\left(\left(\frac{n+1}{n}\right)\frac{u(\langle T\rangle )}{u(T_{p})}\left(T_{p}-T_{max}^{\left(nucl\right)}\right)\right).$$

Here, 〈T〉 obeys the condition $T_{max}^{nucl}\leq \langle T\rangle \leq T_{p}$. Setting 〈T〉 in u(〈T〉) approximately equal to $\langle T\rangle =T_{max}^{\left(nucl\right)}+\left(1/2\right)\left(T_{p}-T_{max}^{\left(nucl\right)}\right)$, we obtain a simple first estimate for the dependence of the change in the peak temperature in overall crystallization. In any of these relations, the crystallization peak temperature increases with increasing heating rate and is determined widely by the dependence of the growth rate on temperature and, in particular, by the value of the activation energy for diffusion, $E_{D}$ (cf. Equations (36) and (37)).

For q→0, Equation (69) predicts $T_{p}=T_{max}^{\left(nucl\right)}$, and from Equations (71) and (72), we arrive at the dependence $(\partial T_{p}/\partial \ln q)≅0$ in this limit. Both limiting conditions are fulfilled in the dependence of $T_{p}$ on heating rate obtained numerically and shown in Figure 9. Accounting for deviations from metastability, then, in particular, the maxima of the nucleation rates also become dependent on the heating rate. Qualitatively, it can be stated that the maxima of the nucleation rate are shifted to higher values, and for this reason, the crystallization peak temperatures are also located at higher temperatures under the same heating rates.

An alternative description of the dependence of the crystallization peak temperature, $T_{p}$, on the rate of change in temperature can be obtained in the following way. For that purpose, we note that Equation (64) can be rewritten (for $T=T_{p}$) as

(73) $$\alpha_{n}\left(T_{p}\right)=1-\exp\left[-Y_{n}\left(T_{p}\right)\right],$$

(74) $$Y_{n}\left(T_{p}\right)=\frac{\omega_{n}}{q^{n+1}}J\left(T_{max}^{\left(nucl\right)}\right)\Delta T^{\left(nucl\right)}{\left(u\left(T_{max}^{\left(growth\right)}\right)\right)}^{n}{\left(\int_{T_{max}^{\left(nucl\right)}}^{T_{p}}\frac{u(T)}{u\left(T_{max}^{\left(growth\right)}\right)}dT\right)}^{n}.$$

Taking the *n*th root of both sides of the second of these equations, we generally obtain

(75) $$\int_{T_{max}^{\left(nucl\right)}}^{T_{p}}\frac{u(T)}{u\left(T_{max}^{\left(growth\right)}\right)}dT=Aq^{\frac{n+1}{n}}$$

with

(76) $$A={\left(\frac{Y_{n}\left(T_{p}\right)}{\omega_{n}J\left(T_{max}^{\left(nucl\right)}\right)\Delta T^{\left(nucl\right)}}\right)}^{1/n}\frac{1}{u\left(T_{max}^{\left(growth\right)}\right)}>0.$$

Computing the derivative with respect to the heating rate *q* and accounting for the dependence $T_{p}=T_{p}\left(q\right)$ results in

(77) $$\frac{dT_{p}}{dq}=A\left(\frac{n+1}{n}\right)q^{1/n}\left(\frac{u\left(T_{max}^{\left(growth\right)}\right)}{u(T_{p})}\right)$$

or

(78) $$\frac{dT_{p}}{dq}=\left(\frac{n+1}{n}\right){\left(\frac{qY_{n}\left(T_{p}\right)}{\omega_{n}J\left(T_{max}^{\left(nucl\right)}\right)\Delta T^{\left(nucl\right)}}\right)}^{1/n}\frac{1}{u\left(T_{p}\right)},$$

respectively, (cf. Equation (62)),

(79) $$\frac{dT_{p}}{dq}=\frac{n+1}{n}{\left(\frac{Y_{n}\left(T_{p}\right)}{\omega_{n}N\left(q\right)}\right)}^{1/n}\frac{1}{u\left(T_{p}\right)}.$$

In the analysis, it was assumed that $Y_{n}$ depends only slightly on $T_{p}$ as also performed in the derivation of Equation (72).

Equations (78) and (79) supply us with a straightforward description of the dependence of the crystallization peak temperature on heating rate and the main factors determining it, and the total number of supercritical clusters formed and their growth rate. However, one has to take into account in the evaluation of this relation that $T_{p}=T_{p}\left(q\right)$, respectively, $q=q(T_{p})$ holds, and N(q) is also not known in advance. In addition, in line with Equations (36) and (37), the growth rate is a complex function of temperature containing two exponential terms. Consequently, an analytical determination of the dependence $T_{p}=T_{p}\left(q\right)$ by this relation is, in general, not possible without further detailed considerations.

In the limit of low temperatures, when the growth rate can be described by $u≅D/4d_{0}$, Equation (78) can be approximated by

(80) $$\frac{dT_{p}}{dq}=\left(\frac{n+1}{n}\right){\left(\frac{qY_{n}\left(T_{p}\right)}{\omega_{n}J\left(T_{max}^{\left(nucl\right)}\right)\Delta T^{\left(nucl\right)}}\right)}^{1/n}\frac{4d_{0}}{D\left(T_{p}\right)}.$$

In this limit, the dependence of the crystallization peak temperature on the heating rate is widely determined by the diffusion coefficient, Equation (36), and the activation energy for diffusion, $E_{D}$. However, even for this case, an integration of this equation or the equivalent to it relation,

(81) $$\int_{T_{max}^{\left(nucl\right)}}^{T_{p}}\exp\left(-\frac{E_{D}}{k_{B}T}\right)dT=C\int_{0}^{q}q^{1/n}dq,$$

$$C=\left(\frac{n+1}{n}\right){\left(\frac{Y_{n}\left(T_{p}\right)}{\omega_{n}J\left(T_{max}^{\left(nucl\right)}\right)\Delta T^{\left(nucl\right)}}\right)}^{1/n}\frac{4d_{0}}{D_{0}\left(T_{p}\right)},$$

in the limits from $\left(T_{max}^{\left(nucl\right)},T_{p}\right)$, respectively, (0,q) seems to be possible only numerically.

With the approximation

(82) $$\int_{T_{max}^{\left(nucl\right)}}^{T_{p}}\exp\left(-\frac{E_{D}}{k_{B}T}\right)dT≅\exp\left(-\frac{E_{D}}{k_{B}T_{d}}\right)\left(T_{p}-T_{max}^{\left(nucl\right)}\right)$$

we obtain an analytical relation for the dependence $T_{p}=T_{p}\left(q\right)$ of the form,

(83) $$T_{p}≅T_{max}^{\left(nucl\right)}+\frac{3}{4}Cq^{4/3}\exp\left(\frac{E_{D}}{k_{B}T_{d}}\right),$$

being in, at least, qualitatively good agreement with the respective curve for overall crystallization in a metastable liquid given in Figure 9. The account of the effects of thermodynamic factors on the growth rate may lead to significant variations in the respective dependencies as is evident from the curves for the overall crystallization of melts not being in metastable equilibrium as also given in the figure mentioned.

Note also that in [63,64] it was shown experimentally that at sufficiently high heating rates the crystallization peak temperature may become independent of the heating rate after an initial increase; instead, as shown here in Figure 9, a plateau is reached. Such behavior obviously requires the incorporation of additional factors such as, eventually, athermal nucleation into the description via the JMAK equation. This problem will be analyzed in detail in a future study.

### 4.3. Crystallization Peak Temperature in Cooling as a Function of the Cooling Rate: Heterogeneous Nucleation

Considering homogeneous nucleation in cooling starting at the melting temperature, $T_{m}$, the situation is quite different when compared to heating. Perceptible nucleation occurs only at low temperatures, and the clusters formed in this range already cannot grow significantly in further cooling. However, for heterogeneous nucleation, the situation is quite different [66,67]. Here, for cooling, we can have a scenario that is quite similar to that discussed for homogeneous nucleation in heating. Indeed, assuming that all heterogeneous nucleation sites become active immediately at the beginning of the cooling process, Equations (23) and (60) result in

(84) $$\alpha_{n}\left(t\right)=1-\exp\left(-Y_{n}\left(t\right)\right),\;Y_{n}\left(t\right)=\omega_{n}N_{het}\left(0\right){\left(\int_{0}^{t}u\left(t^{\prime \prime }\right)dt^{\prime \prime }\right)}^{n}.$$

With

(85) $$T=T_{m}-\left|q\right|t,$$

we obtain

(86) $$\alpha_{n}\left(T\right)=1-\exp\left(-Y_{n}\left(T\right)\right),\;Y_{n}\left(T\right)=\omega_{n}\frac{N_{het}\left(0\right)}{{\left|q\right|}^{n}}{\left(\int_{T}^{T_{m}}u\left(T^{\prime }\right)dT^{\prime }\right)}^{n}.$$

The analysis of these relations can now be performed practically in an identical way as performed in the previous section for overall crystallization in heating. Instead of Equation (69), we can now utilize the relation

(87) $${\tilde{Y}}_{n}=\frac{1}{{\left|q\right|}^{n}}{\left(\int_{T_{p}}^{T_{m}}u\left(T^{\prime }\right)dT^{\prime }\right)}^{n}=\mathrm{constant}.$$

Similarly to Equations (70)–(72), we arrive at

(88) $$\frac{dT_{p}}{d|q|}=-\frac{n}{\left|q\right|}\frac{1}{u(T_{p})}\int_{T_{p}}^{T_{m}}u\left(T\right)dT=-\frac{n}{\left|q\right|}\frac{u(\langle T\rangle )}{u(T_{p})}\left(T_{m}-T_{p}\right).$$

Here, 〈T〉 obeys the condition $T_{p}\leq \langle T\rangle \leq T_{m}$. For |q|→0, we have $T_{p}=T_{m}$ and $dT_{p}/d\ln\left|q\right|=0$ again. For zero cooling rates, the peak temperature should be located at $T=T_{m}$ in the present case. This necessary condition of the validity of the approach is, consequently, fulfilled again.

Similarly to Equations (75)–(76), we may also write

(89) $$\int_{T_{p}}^{T_{m}}u\left(T\right)dT=\left|q\right|{\left(\frac{Y_{n}\left(T_{p}\right)}{\omega_{n}N_{het}\left(0\right)}\right)}^{1/n},$$

resulting, in comparison to Equation (79), in

(90) $$\frac{dT_{p}}{d|q|}=-{\left(\frac{Y_{n}\left(T_{p}\right)}{\omega_{n}N_{het}\left(0\right)}\right)}^{1/n}\frac{1}{u(T_{p})}.$$

In the computations, $Y_{n}\left(T_{p}\right)≅$ constant was assumed again. The crystallization peak temperature decreases with increasing cooling rate. The dependence $T_{p}=T_{p}\left(|q|\right)$ is determined widely by the number of supercritical crystallites formed by heterogeneous nucleation and the dependence of their growth rate on temperature. Equation (90) supplies us in this way with the possibility to estimate the number of heterogeneously formed supercritical nuclei in the system under consideration if $T_{p}=T_{p}\left(|q|\right)$ is experimentally determined. Note also that in cooling under the conditions considered, the growth rate is determined not only by the activation energy for diffusion but also by thermodynamic factors. In this way, not just one exponential term but more than one determine the dependence $T_{p}=T_{p}\left(|q|\right)$.

## 5. Summary of Results and Discussion

The results of the present analysis can be summarized as follows: (i) Relations in the form of Equation (14) and their consequences, such as in Equations (15)–(19), are not applicable any more to the description of the overall crystallization kinetics if relaxation processes of the glass-forming liquid to the respective metastable equilibrium state have to be accounted for. The analysis has to be performed based on the general relations given by Equations (10) and (11). The same statement is valid for heterogeneous nucleation since the growth rates become dependent on time. In line with the latter two relations, the degree of crystallization at some given time is not a function of temperature but a function of combinations of the nucleation and growth rates. (ii) Under both isothermal conditions and cooling and heating with some given rate of change in temperature, the overall crystallization processes may significantly depend on the degree of deviation of the liquid from equilibrium. This effect is more pronounced for nucleation as compared to growth as far as the commonly observed location of the maxima of nucleation and growth rates is realized for the system under consideration. The maximum of the nucleation rate for homogeneous nucleation is commonly located at lower temperatures as compared to the maximum of the growth rates. For this reason, nucleation proceeds predominantly in temperature ranges where deviations from metastable equilibrium are of significant importance. This effect is less pronounced for growth processes proceeding at higher temperatures. (iii) For both cooling and heating, the thermodynamic driving force is, as a rule, larger as compared with the case when the liquid is in the corresponding metastable equilibrium state. However, the surface tension behaves differently; it is larger for cooling processes, and in heating may become smaller when compared with the value corresponding to nucleation in a metastable liquid. These differences in the surface tension and the thermodynamic driving force of crystallization as compared with the case that the liquid is in a metastable state are the origin of the hysteresis effects in crystal nucleation discussed here. They may result in nucleation flashes and, more generally, in the “flare-up” of fluctuations under heating as discussed first long ago by Porai-Koshits, Mazurin, and coworkers and noted also by Davis [69,70,71,72] (for details see also [73,74]). These effects are of particular significance if driving force and surface tension deviate significantly for cooling and heating, i.e., if deviations of the liquid from metastable equilibrium considerably affect the thermodynamic driving force and surface tension. The mechanism of nucleation and growth, as outlined here, gives in this way a principally new method for the treatment of hysteresis effects in overall crystallization under cooling and heating, and of the nucleation flashes in heating in particular. (iv) The analysis of nucleation–growth processes at changing temperatures is a much more complex problem as compared to the theoretical treatment of this process under isothermal process conditions. Extending the previously obtained results in [13], the average time of formation of the first supercritical nucleus in cooling and heating was specified. These results allow one to determine the time and temperature when the nucleation–growth processes become of importance. The crystallization peak is determined, then, as the result of nucleation and the subsequent growth of the supercritical clusters proceeding after the first nucleus has been formed. Different approaches have been developed in the past to describe this overall crystallization process analytically [7,8,9,10,11,12,13,14,15,16,17,18,19,20]. In most of these attempts, the degree of crystallization or the location of the crystallization peak temperature is expressed as some function of temperature introducing some activation energy chosen in such a way that the degree of crystallization and the crystallization peaks are specified more or less correctly. From a mathematical point of view, the degree of crystallization is then described as a function of temperature. However, such treatment is, from a principal point of view, not correct and can only be an approximation. Indeed, the degree of overall crystallization is, as evident from Equations (10) and (11) or Equation (61), a functional of nucleation and growth rates. As evident from these relations, several parameters, such as the work of critical cluster formation, the thermodynamic driving force for cluster growth, and the activation energy for diffusion, significantly affect the values of the functionals. Employing the differences in the locations of the maxima of the nucleation and growth rates in terms of the dependence of temperature, in the present approach, the degree of crystallization could be expressed as a functional only of the growth rates. This method leads to adequate expressions for the dependence of the crystallization peaks on the rates of change in temperature as shown in the present paper. In line with the approximation, the effective activation energy for diffusion is the dominant parameter determining the mentioned dependence. As shown, this approach is appropriate for dominant homogeneous nucleation in heating and dominant heterogeneous nucleation in cooling. The possibility of modeling the crystallization peak temperature in heating via the Kissinger equation can be correlated in this way with the validity of the model employed by us for heating (and similarly for heterogeneous nucleation in cooling). In general, attempts to model the crystallization peak temperature with the aid of the Kissinger equation are quite problematic [20]. In all fairness, Kissinger proposed his equation to be used for describing the kinetics of decomposition [17] and never mentioned the possibility of its use for the crystallization kinetics. Nevertheless, the equation has been applied broadly to analyze the crystallization kinetics occurring on heating as well as on cooling. The major problem with such applications is that the Kissinger equation was derived for the processes whose rate depends on temperature in accord with the Arrhenius equation. The latter represents an exponential function with a temperature-independent parameter called the activation energy. In reality, the temperature dependence of the crystallization rate is much more complex, as is evident from the general relations, Equations (10) and (11). Per Equations (27) and (35)–(37), it is represented by a combination of two exponential functions whose parameters ($E_{D}$, $W_{c}$, σ, and Δμ) are temperature-dependent. Even utilizing the simplified model approach as advanced here, two temperature-dependent exponential terms remain in the description. As a result, the Kissinger equation can work only as an approximation and only in the case when the crystallization kinetics is dominated by the rate of diffusion. In principle, this situation can be realized when crystallization occurs at temperatures far below $T_{m}$, i.e., when Δμ becomes large enough to turn the bracketed term in Equation (37) into one. Such conditions can be at least partially met when crystallization is performed by heating glassy samples but not when cooling the melts. The problem with crystallization upon cooling is exacerbated further by the fact that the derivations used for obtaining the Kissinger equation make it inapplicable to the conditions of cooling [19]. (v) A variety of additional factors may have to be incorporated into the present theory as reviewed briefly in Section 2.3. They may lead to quantitative modifications of the results. However, the general conclusions concerning the necessity to account for deviations in the state of the liquid from metastable equilibrium for a correct description of the kinetics of overall crystallization will remain, as we deeply believe, unchanged. The approach to such a quantitatively more correct treatment is a task that we consider will be solved in future analysis.

## Data Availability

Not applicable.

## Appendix A. Characteristic Times of Relaxation and Crystallization: Some General Considerations

A variety of specific features of crystal nucleation and growth as compared with other types of first-order phase transformations are caused by the possibility that the liquid may not crystallize with increasing deviations in its state from equilibrium but may be transferred into a glass. One particular problem in this connection is found in the analysis of possible ways of resolution of the Kauzmann paradox [54], i.e., in the discussion of the question as to whether liquids under cooling may or may not reach metastable equilibrium states with lower volume density of entropy as compared to the crystal. The temperature at which the entropy densities of liquid and crystal become identical is denoted today as the Kauzmann temperature, $T_{K}$. As one of the mechanisms preventing such a seemingly paradoxical situation, Kauzmann suggested that intensive crystallization takes place slightly above this temperature (at the pseudo-spinodal curve as was denoted by him), precluding the realization of such states.

Kauzmann himself also originally denoted the temperature of the occurrence of the pseudo-spinodal as $T_{k}$ and also discussed different possibilities of the location of the, defined by him, temperature $T_{k}$ as compared to the glass transition temperature, $T_{g}$, and its consequences ([54], pp. 247–248 (I would like to thank Gyan P. Johari for reminding me of this point)). In particular, he wrote:

*“Let us denote by $T_{k}$ the temperature at which the two kinds of barriers become equal. $T_{k}$ may be above or below the glass–transformation point, $T_{g}$, as defined in terms of the ’conventional’ duration of an experiment. If $T_{k}$ is above $T_{g}$, it will be impossible for the liquid to be studied as a liquid at temperatures between $T_{k}$ and $T_{g}$, other than by experiments of much shorter duration than the ’conventional’ one, since it will crystallize spontaneously during any experiments requiring more time than the average time between simple molecular jumps. According to the concepts presented in the previous part of this paper, adequate measurements under these circumstances would result in glass-like properties for the liquid. On the other hand, if $T_{k}$ is below $T_{g}$, it is still possible to distinguish between a glassy state and a true metastable liquid between $T_{g}$ and $T_{k}$. But below $T_{k}$ no such distinction is possible; the glass is then the only experimentally attainable form of the liquid …Accordingly, provided the free energy barriers vary with the temperature in the way that we have postulated, it is not permissible to extrapolate the curves in figures 3 to 6 indefinitely below $T_{g}$ and to infer thereby the existence of a ’thermodynamic’ glass–like transition”.*

In this discussion [54], Kauzmann distinguished two sources for metastability in glass-forming systems: metastability connected with the work of critical crystal cluster formation required for liquid crystal phase transitions and metastability caused by the necessity to form an activated complex in order to realize transport processes such as diffusion or viscous flow. Discussing the dependence of both types of activation barriers on temperature, he wrote:

*“Suppose that when the temperature is lowered a point is eventually reached at which the free energy barrier to crystal nucleation becomes reduced to the same height as the barriers to the simpler motions…At such temperatures the liquid would be expected to crystallize just as rapidly as it changed its typically liquid structure to conform to a temperature or pressure change in its surroundings…There are good theoretical reasons for believing in the existence of such a ‘pseudo-critical temperature’”* ([54], pp. 220 and 247).

Kauzmann distinguishes such pseudo-critical states from critical points or states along the spinodal curve noting:

*“In the past there has been a considerable amount of speculation concerning the existence of a critical point between crystalline and liquid states analogous to the critical point between liquids and gases. No experimental evidence for or against such a critical point has ever been found [75], though there is reason to believe that none is possible (Bernal [76]; but see Frenkel … [77]). It is apparent, however, that the behavior with which we are here concerned has a certain similarity to the behavior at a critical point in that here, as at a true critical point, the free energy barrier between the crystal and the liquid disappears. On the other hand, there is a fundamental difference in that the two states do not really merge and their free energies are decidedly different …, so that one cannot go reversibly from the one state to the other without a normal phase change”* ([54], p. 248).

Meanwhile, the absence of a spinodal in one-component melt crystallization was established by Skripov and Baidakov based on a thorough analysis of experimental data, first in [78] and reconfirmed in [4,79]. The absence of the spinodal is connected with the absence of an equilibrium critical point on the liquid–solid coexistence curve as formulated first based on symmetry concepts by Landau [80,81]. Employing the basic concepts of classical nucleation theory (CNT) utilizing the thermodynamic theory of heterogeneous systems as developed by Gibbs [82], the conclusion of the absence of a spinodal in melt crystallization can be extended to multi-component systems as performed in [43]. In brief, according to CNT, the steady-state nucleation rate, *J*, can be expressed via the work of critical cluster formation, $W_{c}$, as

(A1) $$J=J_{0}\exp\left(-\frac{W_{c}}{k_{B}T}\right),\;W_{c}=\frac{1}{3}\sigma A_{c},\;R_{c}=\frac{2\sigma }{\Delta g}.$$

The steady-state nucleation rate, *J*, is equal to the average number of supercritical crystal clusters formed per unit time in a unit volume of the melt; $J_{0}$ is a kinetic prefactor determined by the kinetics of aggregation. In Equation (A1), $k_{B}$ is the Boltzmann constant, and *T* the absolute temperature. Assuming a spherical shape (choosing the surface of tension as the dividing surface), the surface area of the critical cluster is given by $A_{c}=4\pi R_{c}^{2}$, where $R_{c}$ is the critical cluster radius. It is determined by the ratio of the surface tension, σ, and the thermodynamic driving force of crystallization, Δg, as $R_{c}=2\sigma /\Delta g$. The thermodynamic driving force of nucleation in dependence on temperature is given generally in the form [5,6]

(A2) $$\Delta g\left(T,p_{m}\right)=-\int_{T_{m}}^{T}\Delta s\left(T,p_{m}\right)dT.$$

Here, $T_{m}$ is the melting or liquidus temperature and $p_{m}$ the melting pressure corresponding to it. Approximately, it may be written as

(A3) $$\Delta g\left(T,p_{m}\right)≅\Delta h_{m}\left(1-\frac{T}{T_{m}}\right)\left[1-\frac{\Delta c_{p}}{2\Delta s_{m}}\left(1-\frac{T}{T_{m}}\right)\right].$$

Assuming validity of the Stefan–Skapski–Turnbull relation [5,6], for moderate deviations from equilibrium, the surface tension can be approximately expressed by [43]

(A4) $$\frac{\sigma (T,p_{m})}{\sigma (T_{m},p_{m})}≅\frac{T\Delta s(T,p)}{T_{m}\Delta s_{m}\left(T_{m},p_{m}\right)}.$$

It may be transformed to

(A5) $$\frac{\sigma (T,p_{m})}{\sigma (T_{m},p_{m})}≅\left(\frac{T}{T_{m}}\right)\left[1-\frac{\Delta c_{p}}{\Delta s_{m}}\left(1-\frac{T}{T_{m}}\right)\right].$$

At the Kauzmann temperature, the thermodynamic driving force approaches a maximum [43], and employing the approximation for the surface tension, the latter quantity tends to zero. Despite the surface tension approaching zero (which may not be correct in a more accurate description), the crystallization of liquids does not exhibit features indicating the possible identity of the liquid and crystal states, as is the case at the critical point and the spinodal in liquid–gas transitions or segregation processes in solutions.

The existence of a spinodal curve requires a simultaneous approach of setting both thermodynamic driving force and surface tension to zero since both phases become indistinguishable here. These conditions are not fulfilled.

A similar point of view was expressed by Kelton and Greer ([83], p. 107) in their discussion of the behavior of the work of critical cluster formation, $W_{c}$, for some model computations, where the latter quantity also tends to zero. They note:

*“The decrease of $W_{c}$ to zero is a failing of this particular model, since there is no point at which the liquid becomes unstable relative to the solid.”*

They then continue:

*“Nonetheless, it does indicate that a properly constructed density-functional model could describe the transition from a nucleation and growth mechanism to a spinodal transformation, which the CNT cannot do”*.

Some eventually possible scenarios in this direction (taking account of the dependence of the bulk state parameters of the critical clusters on supersaturation, crystallization via preferential segregation of the liquid) are discussed in [37].

The second item we would like to analyze is whether a pseudo-spinodal exists, whether its existence results in an intensive increase in the rates of crystallization, or whether the pseudo-spinodal may have some other implications. As is well-known from CNT, the dependence of the steady-state nucleation rate on temperature is determined according to Equation (A1) by the interplay of the thermodynamically based term (proportional to the probability of thermal fluctuations required to form a critical cluster, i.e., $\propto \exp(-W_{c}/k_{B}T)$) and the kinetic term ($J_{0}\propto D=D_{0}\exp\left(-E_{D}/k_{B}T\right)$, where *D* is the diffusion coefficient determining the kinetics of aggregation in crystallization). The first term approaches zero at the melting temperature and (for $W_{c}\neq 0$) in the limit T→0. Under typical conditions, it exhibits a maximum in between both limits as discussed, for example, by Tammann [65] and Turnbull (Figure 2 in [84]). The kinetic term decreases monotonically and significantly with decreasing temperature due to the decrease in the diffusion coefficient or the increase of the viscosity with decreasing temperature. As a consequence, the temperature dependence of the steady-state nucleation rate is characterized by a maximum [66] where the kinetic term (decrease in the diffusion coefficient) starts to overestimate the increase in the thermodynamic term. This maximum is generally found to be located near to the glass transition temperature, $T_{g}$, according to the definition by Tammann [38], and is equal to $T_{g}≅\left(2/3\right)T_{g}$ for a variety of glass-forming melts and equal to $T_{g}≅\left(1/3\right)T_{m}$ for metallic glass-forming alloys [5,6,54]. There are no indications in Equation (A1) of a renewed dramatic increase in the nucleation rate at temperatures significantly below this maximum, which could be correlated with a pseudo-spinodal as suggested by Kauzmann. A huge amount of experimental data on crystallization proves that such a type of dependence of the steady-state nucleation rate on temperature is not an exception but the rule, and, experimentally, no exceptions are observed.

As noted earlier, Kauzmann introduced his hypothesis of the existence of a pseudo-spinodal in melt crystallization based on the assumption of the possible near equality of the activation energies for diffusion and crystal nucleation at low temperatures, respectively, the approach of characteristic timescales for relaxation, $\tau_{R}$, and crystallization, correspondingly, i.e., $\tau_{R}≅\langle \tau \rangle$. Following Kauzmann’s original argumentation, the relation of these characteristic timescales has been discussed intensively (for an overview, see [43]). As the characteristic timescale for crystallization, commonly, the average time, 〈τ〉, of the formation of the first supercritical nucleus is chosen as identified by different authors either with the time lag, $\tau_{ns}$ for steady-state nucleation at the given temperature (e.g., [85]) or, alternatively, as the time required to form the first supercritical crystallite under steady-state conditions, ${\langle \tau \rangle }_{ss}=1/\left(J_{ss}V\right)$ (e.g., [55]). Note that the second approach, also actually followed by Kauzmann, does not give a unique definition of the pseudo-spinodal. The result depends significantly on the volume, *V*, of the liquid. Moreover, the average time of formation of the first supercritical nucleus, 〈τ(T)〉, is determined, in general, as the sum of both these terms. As an approximate analytical estimate (see Equation (11) in [41]), we can express 〈τ(T)〉 as

(A6) $$\langle \tau \left(T\right)\rangle ≅\tau_{ns}\left(T\right)+{\langle \tau \left(T\right)\rangle }_{ss},\;{\langle \tau \left(T\right)\rangle }_{ss}=\frac{1}{J_{ss}\left(T\right)V},$$

(A7) $$\tau_{ns}=\frac{\omega }{2}\left(\frac{k_{B}T}{\sigma d_{0}^{2}}\right)\left(\frac{R_{c}^{2}}{D\tau_{R}}\right)\tau_{R}.$$

The numerical factor ω varies in the range 1≤ω≤4 depending on the method employed in the derivation of Equation (A7). Above the decoupling temperature, $T_{d}$, the product $D\tau_{R}$ becomes widely independent of temperature since, in this range, the Stokes–Einstein–Eyring relation may be employed; below $T_{d}$, it cannot. Numerical estimates accounting, as necessarily required, for the decoupling of diffusion and relaxation lead to the conclusion that the condition for the pseudo-spinodal curve, $\langle \tau \rangle ≅\tau_{R}$, as formulated by Kauzmann, is generally fulfilled near to the glass transition temperature.

This result can also be given an alternative verification. For crystal nucleation in viscous liquids, the effective value of the stress parameter ε, the elastic energy per particle of the crystal phase, is determined by the interplay of stress evolution (caused by the formation of a crystallite) and stress relaxation accompanying this process. Assuming, as carried out here, that relaxation is described by Maxwell’s relaxation law, the effective value of ε for a crystallite of critical size is given by

(A8) $$\frac{\varepsilon (j_{c})}{\varepsilon_{0}}≅\frac{\tau_{R}}{\tau_{ns}}\left[1-\exp\left(-\frac{\tau_{ns}}{\tau_{R}}\right)\right].$$

As shown in [5,6] and the papers cited there, the effect of elastic stresses on crystal nucleation in glass-forming liquids is determined by the ratio $\tau_{R}/\tau_{ns}$. $\varepsilon_{0}$ is the respective value for crystallization in a Hookean solid. For temperatures sufficiently above the glass transition temperature, elastic stresses can be expected to be of minor importance; for glasses, they have to be of the same order of magnitude as for Hookean solids. Consequently, under a decreasing temperature, the ratio $\tau_{ns}/\tau_{R}$ has to change from zero near to the melting temperature to very large values below the glass transition temperature to appropriately fulfill the mentioned general condition. Consequently, the condition for the occurrence of the pseudo-spinodal as formulated by Kauzmann will be fulfilled as the rule near to the glass transition temperature $T_{g}$. The steady-state nucleation rate has a maximum there but its dependence on temperature is well-reflected by the standard equations of CNT. As shown in [21,23,37] and demonstrated also in the present paper, upon further cooling of the liquid to states below the glass transition temperature, the steady-state nucleation rate is additionally decreased due to the interplay of relaxation and crystal nucleation. Consequently, knowledge of the ratio $\tau_{ns}/\tau_{R}$ is really of significant importance in modeling crystal nucleation but in quite another context compared to that supposed originally by Kauzmann.

## Appendix B. Application of the Lattice-Hole Model in the Computations and Some Possible Generalization of this Model

In this appendix, we present the results of numerical computations modeling the evolution of the degree of overall crystallization, $\alpha_{n}$, for the lattice-hole model considered here assuming steady-state nucleation and growth in three (n=3) independent directions again. The deviations of the state of the liquid from metastable equilibrium are described by Equations (32) and (33). The results are presented in Figure A1 and Figure A2. A comparison with Figure 3 and Figure 4 shows that deviations in the curves occur but they are relatively small. For this reason, we would like to anticipate here a possible generalization of this model leading eventually to more pronounced results.

For the specification of the thermodynamic properties of glass-forming melts, we employ here relations derived from a simple lattice-hole model of liquids discussed in detail in [5,6]. The structural order–parameter is connected in the framework of this model with the free volume of the liquid and defined via the number of unoccupied sites (or holes), $N_{0}$, per mole of the liquid, with each of them having a volume, $v_{0}\left(T,p\right)$, identical to the volume of a structural unit of the liquid at the same values of pressure and temperature. According to this model, the molar volume of the liquid is determined as

(A9) $$V\left(T,p,\xi \right)≅N_{A}v_{0}\left(T,p\right)\left(1+\xi \right),\;\xi =\frac{N_{0}}{N_{A}+N_{0}}≅\frac{N_{0}}{N_{A}}.$$

Here $N_{A}$ is the Avogadro number.

The thermodynamic functions of the system are described in the framework of this model by the sum of contributions resulting from the thermal motion of the molecules of the liquid and, in addition, from the configurational contributions described by the structural order parameter, ξ. The configurational contribution to the volume is given, consequently, by

(A10) $$V_{\xi }≅N_{A}v_{0}\left(T,p\right)\xi .$$

The configurational contribution to the enthalpy, $H_{\xi }$, of one mole of the liquid is described in the framework of this lattice-hole model via the molar heat of evaporation, $\Delta H_{ev}\left(T_{m}\right)$, of the liquid at the melting temperature as

(A11) $$H_{\xi }=\chi_{1}\Delta H_{ev}\left(T_{m},p\right)\xi .$$

$\chi_{1}$ is a parameter that has to be determined appropriately. Experimental data show that the molar heat of evaporation can be expressed for a wide class of liquids in accordance with Trouton’s rule as

(A12) $$\Delta H_{ev}\left(T_{m},p\right)≅\chi_{2}RT_{m}\;\mathrm{with}\;\chi_{2}=20.$$

According to Equation (A11), $H_{\xi }$ is taken as a function of the melting temperature, $T_{m}$. A much more reasonable and better assumption would be to take it as being equal to the respective value at the current temperature. In this way, we may formulate a generalization of Equation (A11) in the form

(A13) $$H_{\xi }=\chi_{1}\Delta H_{ev}\left(T,p\right)\xi .$$

Furthermore, we can make the substitution

(A14) $$\Delta H_{ev}\left(T,p\right)=\Delta H_{ev}\left(T_{m},p\right)+\Delta C_{p}\left(T_{m},p\right)\left(T-T_{m}\right)$$

with

(A15) $$\Delta C_{p}\left(T_{m},p\right)=C_{p}^{\left(vapor\right)}-C_{p}^{\left(liquid\right)}.$$

The configurational contribution to the entropy per mole is described in this model via the conventional mixing term

(A16) $$S_{\xi }=-R\left(\ln\left(1-\xi \right)+\frac{\xi }{1-\xi }\ln\xi \right).$$

With H=U+pV, for the configurational contribution to the internal energy and the Gibbs free energy, we obtain

(A17) $$U_{\xi }=\left[\chi_{1}\left(\chi_{2}RT_{m}+\Delta C_{p}\left(T_{m},p\right)\left(T-T_{m}\right)\right)-pv_{0}\left(T,p\right)\right]\xi ,$$

(A18) $$G_{\xi }=\chi_{1}\left(\chi_{2}RT_{m}+\Delta C_{p}\left(T_{m},p\right)\left(T-T_{m}\right)\right)\xi +RT\left(\ln\left(1-\xi \right)+\frac{\xi }{1-\xi }\ln\xi \right).$$

The equilibrium value of the structural order parameter, $\xi =\xi_{e}$, is determined via the relation ${\left(\partial G_{\xi }/\partial \xi \right)}_{T,p}=0$ resulting in

(A19) $$\frac{{\left(1-\xi_{e}\right)}^{2}}{\ln\xi_{e}}=-\left(\frac{T}{T_{m}}\right)\frac{1}{\chi_{1}\left[\chi_{2}+\frac{\Delta C_{p}\left(T_{m},p\right)}{R}\left(\frac{T}{T_{m}}-1\right)\right]}.$$

It is different from the result employed in the present analysis (Equation (23)) and may lead to a more pronounced effect of deviations of the liquid from equilibrium on crystallization. A detailed analysis is considered to be of interest but out of the scope of the present paper.
